# Discovery of Sennoside A as a Natural *O*-GlcNAcase Inhibitor That Ameliorates Parkinson’s Disease by Restoring EphrinB2 *O*-GlcNAcylation

**DOI:** 10.34133/research.1416

**Published:** 2026-09-09

**Authors:** Guilin Wei, Zhuoan Jin, Hanying Mi, Nan Li, Weiyu Wang, Yunqing Song, Qiang Jin, Guangbo Ge, Yong Liu

**Affiliations:** ^1^ State Key Laboratory of Discovery and Utilization of Functional Components in Traditional Chinese Medicine, Shanghai Frontiers Science Center of TCM Chemical Biology, Institute of Interdisciplinary Integrative Medicine Research, Shanghai University of Traditional Chinese Medicine, Shanghai 201203, China.; ^2^ School of Chemical Engineering, Ocean and Life Sciences, Dalian University of Technology, Panjin 124221, China.; ^3^ Jiangxi Provincial Key Laboratory of Natural Microbial Medicine Research, School of Pharmacy, Yichun University, Yichun 336000, China.

## Abstract

Down-regulated *O*-linked β-*N*-acetylglucosamine modification (*O*-GlcNAcylation) has been implicated in Parkinson’s disease (PD), and restoring *O*-GlcNAcylation levels in the central nervous system via *O*-GlcNAcase (OGA) suppression represents a potential therapeutic strategy for PD intervention. Nevertheless, naturally derived OGA inhibitors remain insufficiently explored, and the molecular mechanisms underlying OGA-inhibition-mediated restoration of *O*-GlcNAcylation to alleviate PD remain poorly defined. Here, a fluorescence-based high-throughput inhibitor screening platform was established and subsequently used to identify sennoside A (SA) as a potent OGA inhibitor from an in-house phytochemical library. As a competitive OGA inhibitor, SA exhibited potent anti-OGA activity both in vitro and in vivo, along with favorable blood–brain barrier permeability and a well-tolerated safety profile. Moreover, SA markedly elevated protein *O*-GlcNAcylation levels, which in turn attenuated neuroinflammatory response and ameliorated key PD-associated pathological and behavioral deficits. Mechanistically, SA directly inhibits OGA activity in neurons and microglia, predominantly enhancing the *O*-GlcNAcylation of EphrinB2 at the Ser40/Thr183 sites and stabilizing EphrinB2. This site-specific modification activates the EphrinB2–EphB4 signaling cascade and subsequently blunts mitogen-activated protein kinase-mediated neuroinflammation. Disruption of EphrinB2 *O*-GlcNAcylation by Ser40A/Thr183A mutation eliminates its neuroprotective function and abolishes the anti-PD effects of SA. Collectively, this work establishes the pharmacological inhibition of OGA as a promising therapeutic strategy for PD and identifies SA as a naturally occurring anti-PD agent that alleviates PD-associated pathological phenotypes by inhibiting OGA-mediated de-*O*-GlcNAcylation of EphrinB2.

## Introduction

Parkinson’s disease (PD) represents the second most prevalent neurodegenerative disorder globally, affecting millions of individuals and conferring a considerable socioeconomic burden [1]. As a progressive neurodegenerative condition, PD is characterized by progressive striatal dopamine (DA) depletion attributable to the gradual degeneration of DA neurons in the substantia nigra pars compacta (SNpc) [2–6]. Despite decades of research, current therapeutic strategies remain predominantly symptomatic, relying on DA replacement therapies that do not halt or reverse the underlying neurodegenerative process [7,8]. Moreover, long-term use of these agents is frequently associated with intolerable side effects and diminishing efficacy [9]. Consequently, there is an urgent and unmet clinical need to identify new disease-modifying therapies capable of intervening in the fundamental pathogenic mechanisms driving PD.

In recent years, posttranslational modifications (PTMs) have emerged as critical regulators of cerebral homeostasis, with *O*-linked β-*N*-acetylglucosamine modification (*O*-GlcNAcylation) attracting particular attention. *O*-GlcNAcylation is a dynamic and reversible PTM that mediates *O*-linked β-*N*-acetylglucosamine (*O*-GlcNAc) to the serine and threonine residues of nucleocytoplasmic proteins [10–12]. Accumulating evidence has linked dysregulated *O*-GlcNAcylation homeostasis to PD pathogenesis, including aberrant α-synuclein aggregation, mitochondrial dysfunction, and neuroinflammation [13]. However, the precise mechanisms by which *O*-GlcNAcylation contributes to PD remain poorly understood, particularly the identity of specific protein targets whose *O*-GlcNAcylation is involved and how such modification regulates disease pathology, which remains largely unexplored. As the sole enzyme responsible for the cleavage of *O*-GlcNAc from proteins, *O*-GlcNAcase (OGA) serves as an indispensable gatekeeper of *O*-GlcNAcylation dynamics [14,15]. Blocking OGA has been shown to increase global *O*-GlcNAcylation levels, thereby modulating downstream signaling pathways and exerting neuroprotective effects [16]. However, the molecular mechanisms underlying OGA-inhibition-mediated restoration of *O*-GlcNAcylation to alleviate PD remain poorly defined, and particularly, the selective modification of key pathogenic proteins and its therapeutic relevance need to be further elucidated. Moreover, genetic ablation of OGA leads to embryonic lethality [17], underscoring the essential role of *O*-GlcNAcylation homeostasis in cell survival and highlighting the need for pharmacological modulation rather than complete enzyme ablation. Although several OGA inhibitors have been developed, including Thiamet G (T-G) and *O*-(2-acetamido-2-deoxy-D-glucopyranosylidene)amino-*N*-phenylcarbamate (PUGNAc), their translational potential has been hampered by suboptimal selectivity, off-target effects, or insufficient bioavailability in the central nervous system [18]. More recently, next-generation OGA inhibitors such as MK-8719 have entered clinical development with favorable pharmacokinetic profiles and high central nervous system penetration. However, their application in PD remains exploratory, and challenges such as synaptic toxicity have also emerged [19]. To date, no safe and potent OGA inhibitor has been successfully advanced into clinical development for the treatment of PD, underscoring a critical unmet need for the discovery of novel, selective, and well-tolerated OGA-targeted therapeutics.

Identification of potent and selective OGA inhibitors ultimately depends on the availability of robust high-throughput screening (HTS) platforms capable of capturing dynamic enzyme activity. This requirement is especially critical in the context of PD, where OGA-mediated *O*-GlcNAcylation homeostasis exhibits spatiotemporal dynamics during pathogenesis, necessitating in situ monitoring and real-time assessment of inhibitory efficacy. Conventional analytical methods (liquid chromatography–mass spectrometry [LC–MS] based, enzyme-linked immunosorbent assay [ELISA], Western blot [WB], etc.) are predominantly single-point assays [20], which fail to capture the dynamics of OGA activity. In contrast, optical substrate-based assays have emerged as powerful tools that enable real-time visualization of enzymatic activity with high spatial and temporal resolution, making them particularly valuable for drug screening and functional studies in complex biological systems [21,22]. An optical substrate capable of tracking OGA activity during PD pathogenesis and treatment would therefore be critical for mechanistic exploration and therapeutic development. However, commercially available probes like *p*-nitrophenyl-GlcNAc and 4-methylumbelliferyl-GlcNAc (4MU-GlcNAc) suffer from poor selectivity and are susceptible to interference from autofluorescence or ultraviolet (UV) absorption [23], making them unsuitable for live-cell or in vivo applications. Furthermore, existing few fluorescent substrates lack thorough selectivity validation against other glycosidases, particularly β-*N*-acetylglucosaminidase (NAG) [24–26]. Critically, no fluorescent probe has yet been applied to assess OGA function in PD or to develop corresponding OGA inhibitors for therapeutic purposes. Natural products, with their unparalleled chemical diversity and documented efficacy in alleviating PD symptoms [27–29], represent an ideal reservoir for discovering such inhibitors. Developing a specific fluorescent substrate to establish an HTS method, identifying natural OGA inhibitors that restore the *O*-GlcNAcylation of key proteins, and validating their therapeutic potential in PD thus constitutes a strategic imperative.

**Fig. 1.**
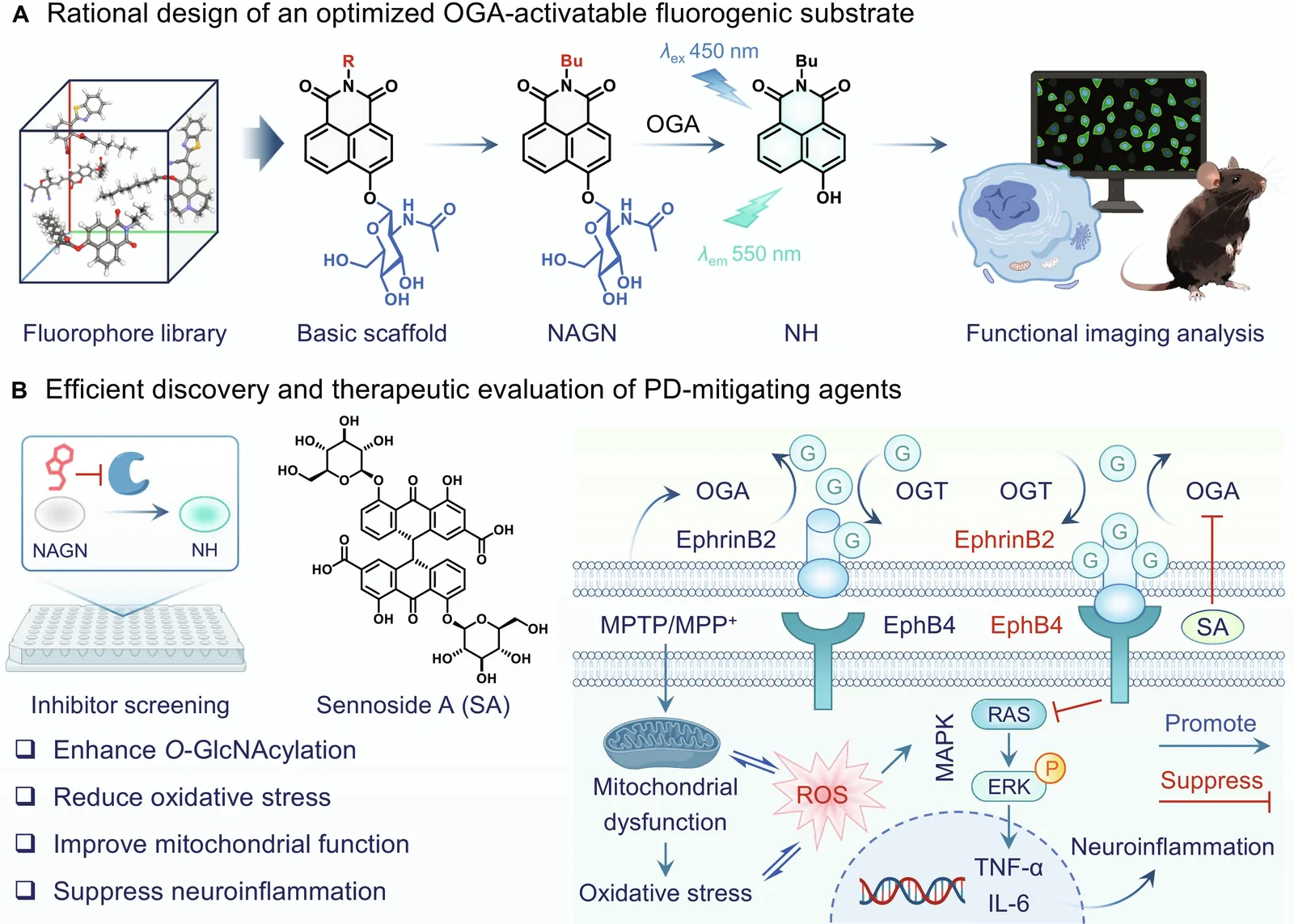
Schematic illustration of a fluorescence-based assay for the discovery of a natural *O*-GlcNAcase (OGA) inhibitor that alleviates Parkinson’s disease (PD) via promoting EphrinB2 *O*-linked β-*N*-acetylglucosamine modification (*O*-GlcNAcylation). (A) Development of an OGA-activatable fluorescent substrate. (B) Discovery and characterization of a potent OGA inhibitor to alleviate PD.

This study aimed to identify potent OGA inhibitors with robust anti-PD effects from natural products and to elucidate the mechanism by which pharmacological inhibition of OGA enhances *O*-GlcNAcylation and confers neuroprotection against PD. To this end, a dedicated fluorogenic substrate for OGA was developed through virtual screening combined with in vitro validation, enabling the establishment of an HTS platform compatible with complex biological systems. Subsequent large-scale screening of an in-house phytochemical library led to the identification of sennoside A (SA) as a potent OGA inhibitor. Further characterization revealed that SA acts as a competitive inhibitor with a favorable safety profile, capable of potently inhibiting OGA activity both in cells and in vivo. Pharmacological evaluation in established PD models further revealed that SA effectively inhibits OGA, elevates *O*-GlcNAcylation levels, and markedly ameliorates PD-associated phenotypes. Finally, multi-omics approaches were used to explore its PD-alleviating mechanism, revealing that SA primarily exerts its PD-mitigating effects by enhancing EphrinB2 *O*-GlcNAcylation and suppressing the mitogen-activated protein kinase (MAPK)-mediated inflammatory pathways (Fig. 1). All these efforts offer a new paradigm to discover novel PD-mitigating agents via targeting OGA-mediated EphrinB2 *O*-GlcNAcylation.

## Results

### Rational design of an OGA-activatable fluorescent substrate

**Fig. 2.**
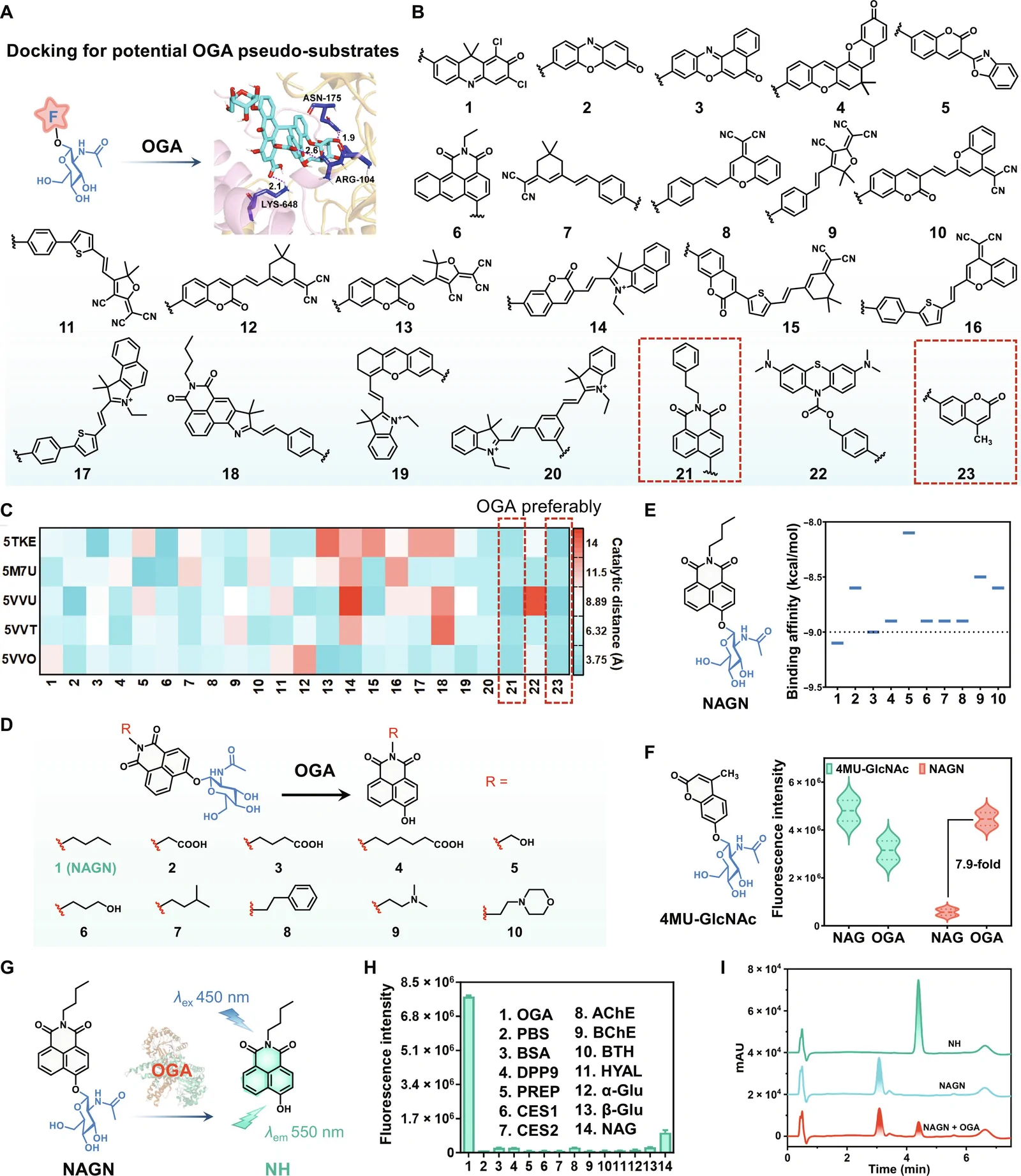
Rational engineering of *O*-GlcNAcase (OGA)-activatable fluorogenic substrates. (A) Docking-based screening for potential fluorophores. (B) The chemical structures of the selected fluorophores. (C) Catalytic distances between the carbonyl group on *N*-acetylglucosamine and the N or D175 amino acid. (D) Chemical structures of 10 naphthalimide-derived candidates. (E) Binding affinities of candidate substrates toward OGA. (F) Fluorescence response of NAGN and 4-methylumbelliferyl-GlcNAc (4MU-GlcNAc) upon OGA and *N*-acetylglucosaminidase (NAG). (G) Schematic diagram of NAGN sensing OGA. (H) Specificity of NAGN. (I) High-performance liquid chromatography–ultraviolet (HPLC–UV) of NH, NAGN, and NAGN + OGA.

It is well established that OGA selectively catalyzes the hydrolysis of *O*-GlcNAc glycosidic linkages [30,31], providing a basis for designing OGA-activatable fluorogenic substrates by incorporating an OGA-specific recognition moiety into a fluorophore scaffold. To screen for the optimal fluorophore, 23 GlcNAc-conjugated fluorophores were collected and individually docked against multiple OGA crystal structures (Protein Data Bank [PDB] IDs: 5VVO, 5VVU, 5VVT, 5M7U, and 5TKE) (Fig. 2A and B). Computational analyses revealed that fluorophores 21 and 23 exhibited the shortest distances between the carbonyl group on the *N*-acetylglucosamine moiety and the catalytic residues within the OGA active site (Fig. 2C and Table S1). Given that fluorophore 23 exhibits a short emission wavelength and may undergo off-target metabolism by other enzymes, fluorophore 21 was chosen for subsequent structural optimization (Fig. 2D). Further molecular docking demonstrated that probe 1 (NAGN) possessed the strongest binding affinity toward OGA, identifying it as the most promising candidate substrate (Fig. 2E and Table S2). Consequently, NAGN was synthesized, with its structural identity characterized by nuclear magnetic resonance (NMR) spectroscopy and high-resolution mass spectrometry (HRMS) (Figs. S1 to S12), and its purity was determined by high-performance liquid chromatography (HPLC) (Fig. S13). The photophysical characteristics of NAGN were systematically investigated, revealing pronounced alterations in both absorption and fluorescence emission profiles upon incubation with OGA (Fig. S14A and B). In vitro assays verified that NAGN possessed superior selectivity over 4MU-GlcNAc, underwent efficient OGA-mediated hydrolysis, and exhibited low cross-activity with homologous NAG (Fig. 2F and G). These findings demonstrate that NAGN serves as a specific and rapid-responding fluorescent substrate for OGA, prompting further investigation into its sensing properties.

### Characterization of NAGN as a specific fluorescent substrate for OGA

Given the abundance of hydrolases and albumin within cells, the hydrolytic profile of NAGN was further evaluated against a panel of these hydrolases and albumin. Notably, none of them exhibited appreciable catalytic activity toward the fluorescent substrate (Fig. 2H). Furthermore, HPLC analysis confirmed that OGA-mediated hydrolysis of NAGN produced a unique fluorescent product, which enabled accurate quantitative detection (Fig. 2I). Moreover, the fluorescence intensity exhibited excellent linear correlations with OGA concentration (0 to 1 μg/ml) and reaction time (within 30 min) (Fig. S14C to F). Additionally, NAGN showed remarkable stability, being unaffected by endogenous amino acids and ions and maintaining stability under physiological pH conditions (Fig. S15A and B). Kinetic analysis revealed that OGA-mediated hydrolysis of NAGN in both purified OGA and human liver cytosol (HLC) followed Michaelis–Menten kinetics with comparable *K*_m_ values, identifying OGA as the major hydrolase for NAGN hydrolysis (Figs. S15C and D and Table S3). Moreover, the kinetic parameters of OGA-catalyzed NAGN hydrolysis determined by HPLC–UV were highly consistent with that acquired via the microplate reader assay (Fig. S15E and Table S3). Together, these results confirm the high specificity of NAGN for OGA and suggest that NAGN is a reliable tool for quantifying OGA activity levels in complex biological systems.

Following the favorable selectivity and sensitivity of NAGN in vitro, its cellular applicability was further evaluated. NAGN and its hydrolytic metabolite NH showed no obvious cytotoxicity at 100 μM across the tested cell lines (Fig. S16). In A549 and SH-SY5Y cells, NAGN treatment resulted in a time-dependent fluorescence signal, which was suppressed by the selective OGA inhibitor T-G and augmented by glucose in a concentration-dependent manner (Fig. S17). In addition, knockout of OGA using small interfering RNA (siRNA) markedly reduced the intracellular green fluorescence (Fig. S18). Together, these results validate the high specificity of NAGN for OGA and demonstrate its applicability for real-time imaging of endogenous OGA activity in living cells. Ex vivo imaging of mouse organs revealed pronounced fluorescent signals across multiple organs, in accordance with the widespread tissue distribution of OGA (Fig. S19A and B). Furthermore, these results agreed well with the OGA activity quantified by HPLC–UV in the same tissue samples (Fig. S19B). Following intraperitoneal administration, marked accumulation of NAGN was detected in the liver and brain at 0.5 and 4 h postinjection (Fig. S19C and D). Collectively, these finds support the utility of NAGN for the multidimensional profiling of OGA activity across biological systems.

### Identification and characterization of SA as a competitive OGA inhibitor

**Fig. 3.**
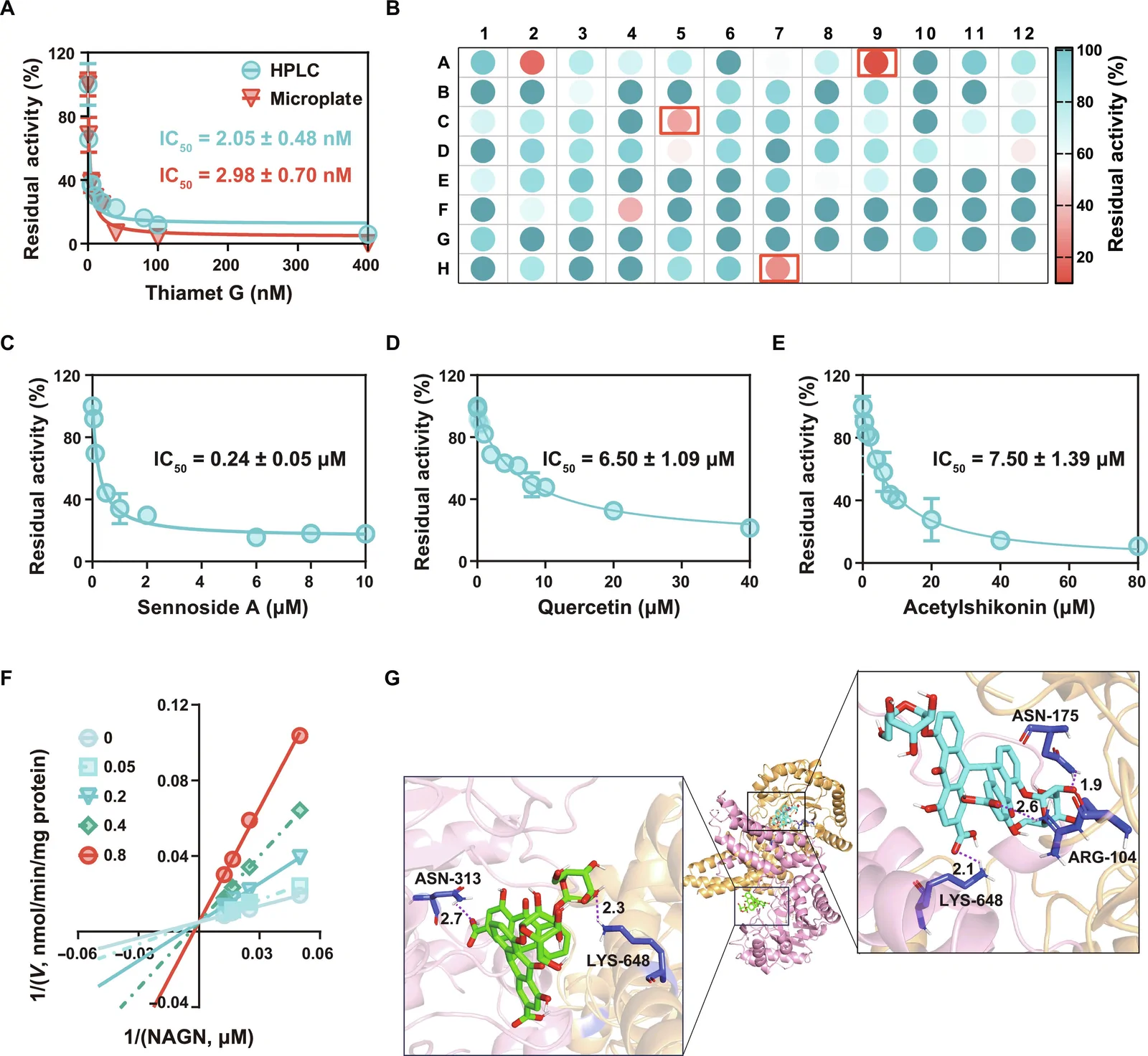
Identification of *O*-GlcNAcase (OGA) inhibitors. (A) The inhibitory effects of Thiamet G on OGA were monitored by both a high-performance liquid chromatography–ultraviolet (HPLC–UV)-based method and a microplate-based method. (B) Inhibitory potentials of tested compounds against OGA. (C to E) Dose–response curves of sennoside A (SA), quercetin, and acetylshikonin against OGA. (F) Inhibition kinetics of SA toward OGA. (G) Docking simulation of SA docked into the catalytic cavity of OGA. All data are expressed as mean ± SD (*n* = 3).

Given the pivotal role of OGA in maintaining *O*-GlcNAcylation homeostasis during PD pathogenesis [32,33], the development of OGA inhibitors holds notable clinical potential. A fluorescence-based inhibitors HTS platform was established by NAGN as the fluorescent substrate, which was subsequently validated using the positive OGA inhibitor T-G (Fig. 3A). Importantly, the IC_50_ values derived from NAGN-based HTS assay were highly consistent with those obtained by the HPLC–UV-based method (Fig. 3A and Table S4), confirming the reliability of this assay. The established platform was then applied to screen an in-house phytochemical library for natural OGA inhibitors. Four candidate compounds, namely, SA, quercetin, acetylshikonin, and echinocystic acid, showed potent inhibitory effects on OGA (Fig. 3B, Fig. S20, and Table S5). Further characterization revealed that SA had the lowest half inhibitory concentration (IC_50_) value of 0.2 μM, indicating the strongest inhibitory potency (Fig. 3C to E and Fig. S21A). We then evaluated the inhibitory effects of SA against other hydrolases, including carboxylesterase 1 (CES1), carboxylesterase 2 (CES2), human pancreatic lipase, prolyl endopeptidase (PREP), and NAG. As shown in Fig. S21B, SA exhibited negligible inhibition against these enzymes, with residual activity remaining above 70%. These results highlight the high selectivity of SA toward OGA over other hydrolases, supporting its utility for studying OGA-mediated biological processes. Crucially, time-dependent inhibition (TDI) assays indicated no progressive enhancement of OGA inhibition with prolonged incubation (Fig. S21C), suggesting that SA acts as a reversible OGA inhibitor. Further kinetic analyses showed that SA strongly inhibited OGA-catalyzed NAGN hydrolysis in a competitive manner, with a *K*_i_ value of 0.14 μM (Fig. 3F and Fig. S21D). Molecular docking simulations further revealed that SA occupies the catalytic site of OGA (Fig. 3G). Further molecular dynamics (MD) simulations (Fig. S22) confirmed that SA stably bound with OGA at the catalytic pocket, offering a structural rationale for the competitive inhibition observed in the kinetic assays. These findings establish SA as a potent and competitive OGA inhibitor, representing a promising lead candidate for the treatment of OGA-associated disorders.

### Neuroprotective effects of SA in PD cellular models

**Fig. 4.**
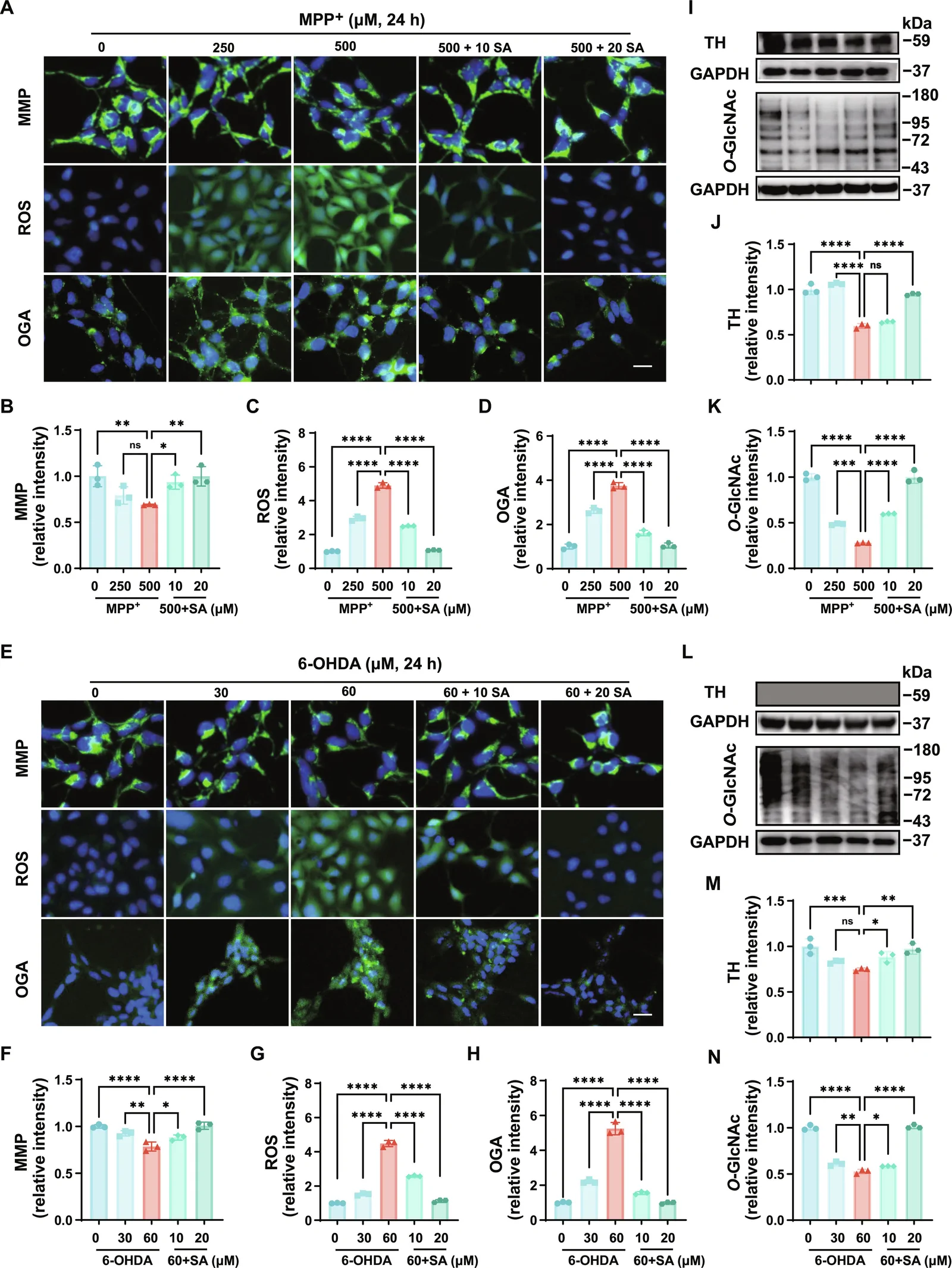
Therapeutic potential of sennoside A (SA) in cellular Parkinson’s disease (PD) models. (A) Fluorescence imaging of mitochondrial membrane potential (MMP), reactive oxygen species (ROS), and *O*-GlcNAcase (OGA) in 1-methyl-4-phenylpyridinium ion (MPP^+^)-challenged SH-SY5Y cells; scale bar = 30 μm. (B to D) Quantification analysis of (A). (E) Fluorescence imaging of MMP, ROS, and OGA in 6-hydroxydopamine (6-OHDA)-challenged SH-SY5Y cells; scale bar = 30 μm. (F to H) Quantification analysis of (E). (I) Western blot (WB) analysis of tyrosine hydroxylase (TH) and protein *O*-GlcNAcylation levels in MPP^+^-challenged SH-SY5Y cells. (J and K) Quantification analysis of (I). (L) WB analysis of TH and protein *O*-GlcNAcylation levels in 6-OHDA-challenged SH-SY5Y cells. (M and N) Quantification analysis of (L). All data are expressed as mean ± SD, *n* = 3; comparisons were analyzed by one-way analysis of variance with Dunnett’s tests. ns, *P* > 0.05; *P < 0.05; **P < 0.01; ***P < 0.005; ****P < 0.001.

To evaluate the therapeutic potential of OGA inhibition mediated by SA in the context of PD, we therefore assessed its capacity to ameliorate disease-relevant phenotypes in well-established cellular PD models. As shown, SA exhibited no cytotoxicity at 20 μM (Fig. S23A) and significantly alleviated 1-methyl-4-phenylpyridinium ion (MPP^+^)-induced neurotoxicity (Fig. S23B). Live-cell imaging revealed that challenge with MPP^+^ or 6-hydroxydopamine (6-OHDA) elevated intracellular OGA activity in a concentration-dependent manner, which was dose-dependently reversed by SA (Fig. 4). Further quantification showed that both neurotoxins markedly increased reactive oxygen species (ROS) generation and disrupted mitochondrial membrane potential (MMP); these impairments were markedly ameliorated by SA cotreatment, with reduced ROS accumulation and restored MMP (Fig. 4A to H). Upon MPP^+^ stimulation in BV2 microglial cells, SA suppressed the transcriptional up-regulation of pro-inflammatory cytokines and augmented the transcript levels of anti-inflammatory cytokines (Fig. S24). WB analysis demonstrated that challenge with MPP^+^/6-OHDA significantly down-regulated tyrosine hydroxylase (TH), a key PD marker [34], whereas SA treatment restored TH expression to near-normal levels (Fig. 4I to M). Notably, cellular *O*-GlcNAcylation levels were significantly reduced in PD model cells but were effectively restored by SA cotreatment (Fig. 4I to N). These observations suggest that SA exerts potent neuroprotective effects in PD cellular models by inhibiting OGA, restoring *O*-GlcNAcylation, and mitigating MPP^+^/6-OHDA-induced neurotoxicity.

### Neuroprotective effects of SA in an MPTP-challenged PD mouse model

**Fig. 5.**
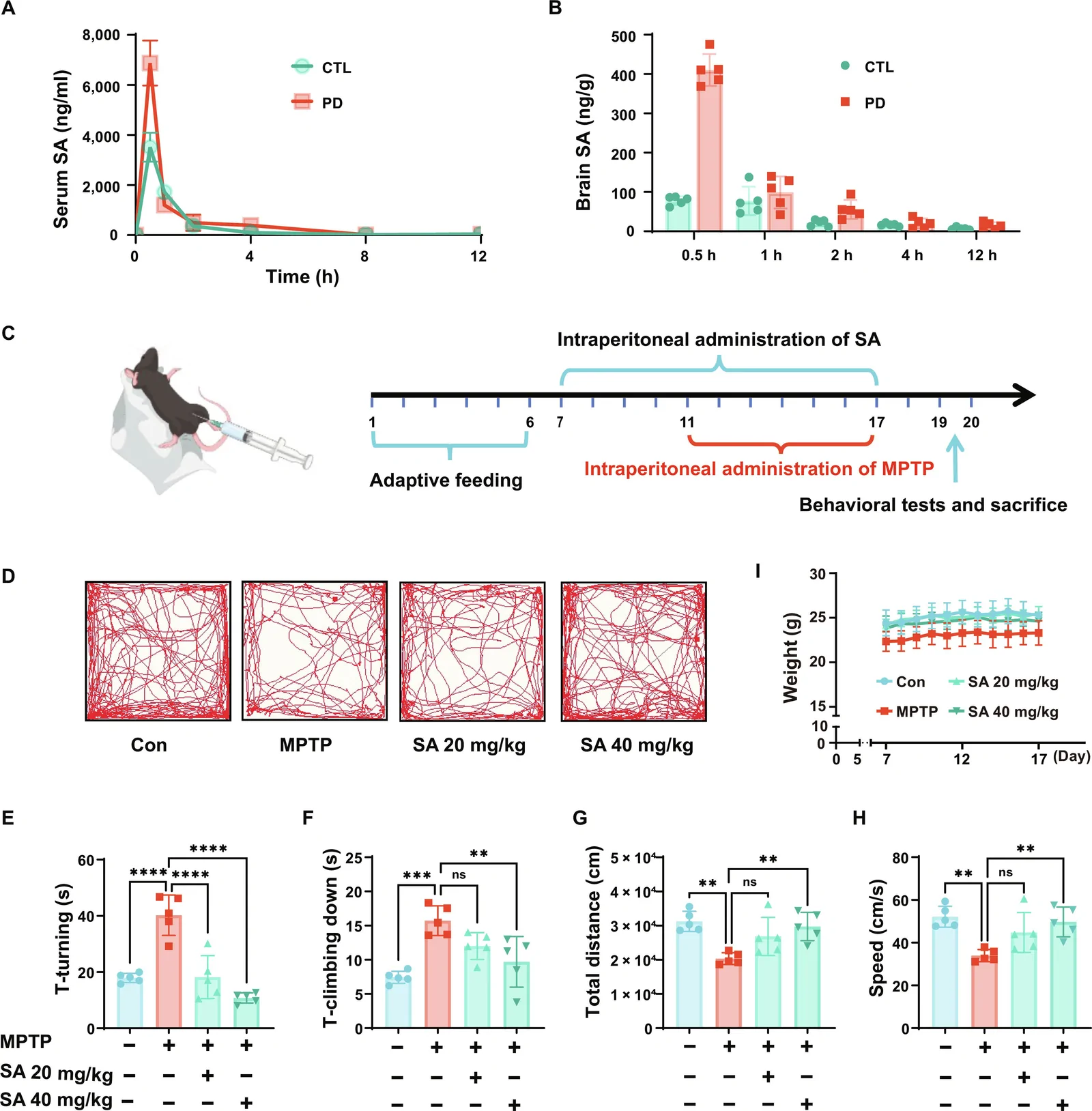
Therapeutic effects of sennoside A (SA) in Parkinson’s disease (PD) mouse models. (A) Plasma concentration–time curves of SA following intraperitoneal injection in normal and PD mice. (B) The exposure levels of SA in the brain at different time points in normal and PD mice. (C) Schematic diagram of animal PD modeling and SA administration. (D) The representative picture of the open-field test. (E) Turning time in the pole test. (F) Time of climbing down in the pole test. (G) Total distance and (H) average speed in the open-field test. (I) Body weight changes in mice. All data are expressed as mean ± SD, *n* = 5; comparisons were analyzed by one-way analysis of variance with Dunnett’s tests. ns, *P* > 0.05; **P < 0.01; ***P < 0.005; ****P < 0.001.

To evaluate the therapeutic potential of SA in vivo, systemic animal studies were performed. Tissue biodistribution assays demonstrated that SA displays widespread tissue exposure and favorable blood–brain barrier (BBB) permeability (Fig. 5A and B and Fig. S25). Notably, brain accumulation of SA was significantly higher in 1-methyl-4-phenyl-1,2,3,6-tetrahydropyridine (MPTP)-challenged PD mice than in control mice, implying compromised BBB integrity during PD pathogenesis that facilitates the central delivery of SA. Tissue imaging further confirmed that SA effectively inhibited OGA activity in situ, supporting its high target specificity in vivo (Fig. S26). In a classical MPTP-challenged PD mouse model (Fig. 5C), MPTP injection caused pronounced motor deficits, which were characterized by significant reductions in total locomotor distance and average speed in the open-field test, as well as marked prolongations in turning and climbing durations in the pole test. SA administration significantly alleviated these MPTP-challenged motor impairments (Fig. 5D to H). Ex vivo fluorescence imaging confirmed that SA effectively suppressed OGA activity in brain tissues, corroborating its on-target action (Fig. S27). Moreover, SA displayed favorable biocompatibility with no obvious body weight loss or histopathological damage in major organs (Fig. 5I and Figs. S28 and S29).

**Fig. 6.**
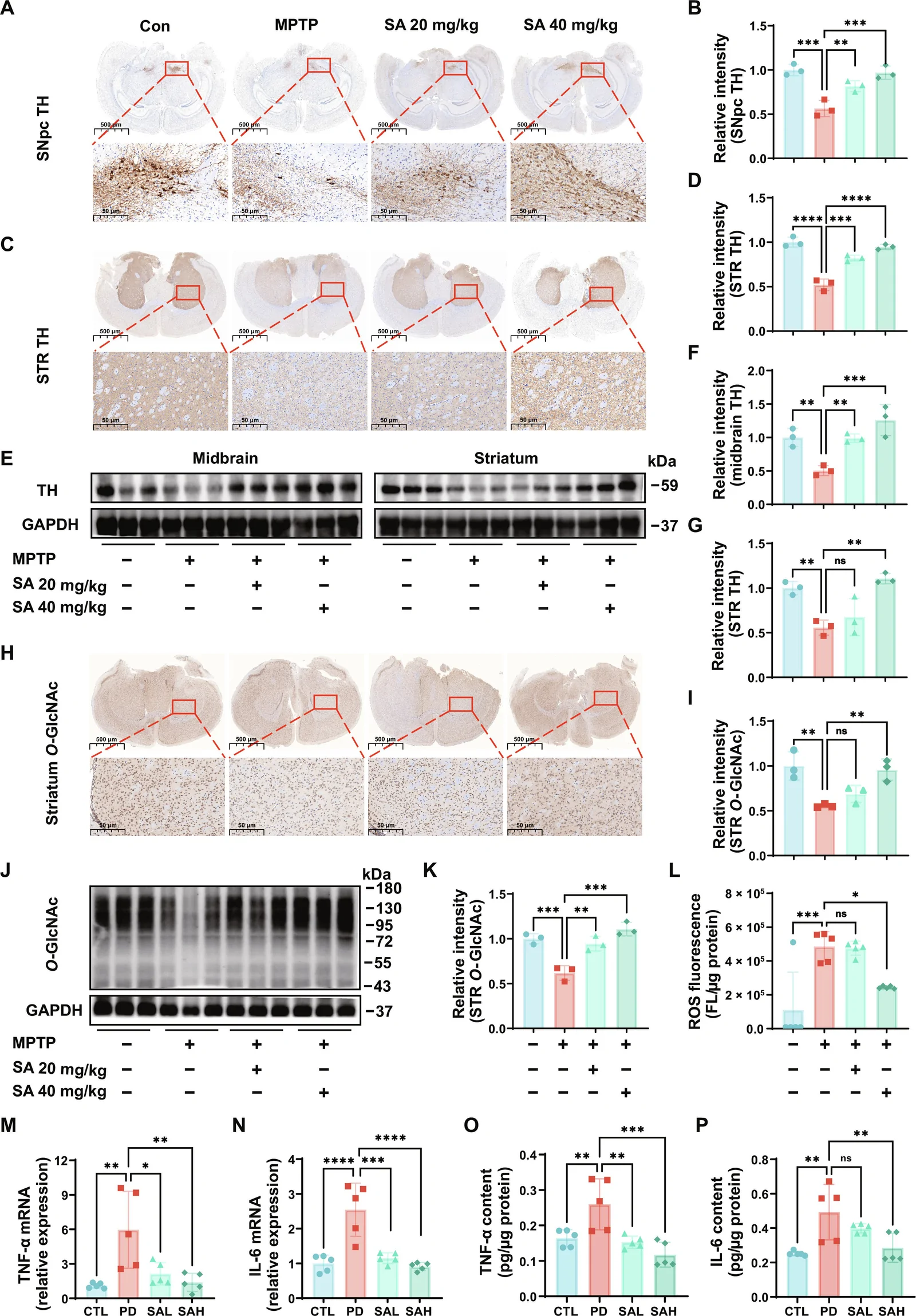
Sennoside A (SA) alleviates dopaminergic neurodegeneration in 1-methyl-4-phenyl-1,2,3,6-tetrahydropyridine (MPTP)-challenged Parkinson’s disease (PD) mice. Immunohistochemistry (IHC) staining and quantification of tyrosine hydroxylase (TH) in the substantia nigra pars compacta (SNpc) (A and B) and striatum (STR) (C and D). (E) Western blot (WB) analysis of TH in the midbrain and STR. (F and G) Quantification of (E). (H and I) IHC diagram and quantification of protein *O*-GlcNAcylation staining in STR. (J and K) WB analysis and quantification of protein *O*-GlcNAcylation levels in STR. (L) Reactive oxygen species (ROS) intensity in different groups of mice. (M and N) Messenger RNA (mRNA) expression of tumor necrosis factor-α (TNF-α) and interleukin-6 (IL-6) in different groups of mouse brain. (O and P) Protein contents of TNF-α and IL-6 in different groups of mouse brain. All data are expressed as mean ± SD, *n* = 3 or 5; comparisons were analyzed by one-way analysis of variance with Dunnett’s tests. ns, *P* > 0.05; *P < 0.05; **P < 0.01; ***P < 0.005; ****P < 0.001.

Immunohistochemical analysis showed that MPTP challenge markedly reduced TH-positive DA neuron numbers in the SNpc and TH-positive nerve fiber density in the striatum (STR), both of which were dose-dependently attenuated by SA administration (Fig. 6A to D). WB analysis further verified that SA restored TH protein expression in the midbrain and STR of MPTP-challenged mice (Fig. 6E to G). Meanwhile, SA treatment reversed the marked reduction in STR *O*-GlcNAcylation levels caused by MPTP challenge (Fig. 6H to K). Moreover, SA significantly attenuated MPTP-induced ROS accumulation and oxidative stress in mouse brain tissues (Fig. 6L). Given the close interplay between ROS production and inflammatory responses [35,36], inflammatory markers were subsequently evaluated following SA treatment. As presented, SA suppressed the release of pro-inflammatory cytokines (tumor necrosis factor-α [TNF-α] and interleukin-6 [IL-6]) and restored the level of the anti-inflammatory cytokine IL-10 in MPTP-challenged mice (Fig. 6M to P and Fig. S30), indicating reduced neuroinflammation. Collectively, these results demonstrate that SA possesses favorable BBB permeability and target specificity toward OGA in vivo, restores STR *O*-GlcNAcylation levels, ad effectively ameliorates MPTP-challenged neuroinflammation in PD mouse models.

### Multi-omics analysis reveals the neuroprotective mechanism of SA in PD

**Fig. 7.**
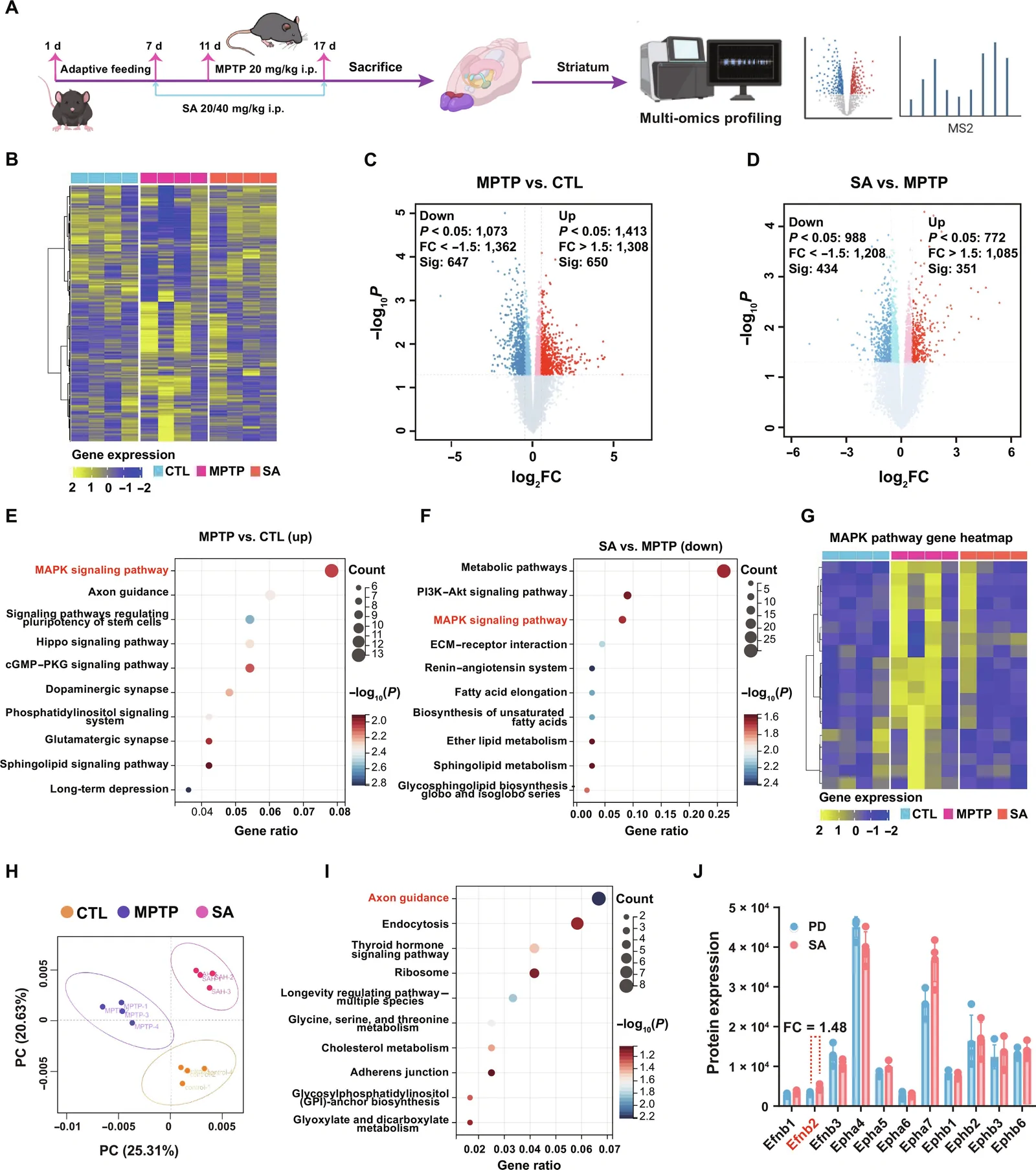
Multi-omics profiling of mouse brains from different groups. (A) Schematic overview of multi-omics analysis revealing the mechanism of sennoside A (SA) in Parkinson’s disease (PD) therapy. (B) Heatmaps displaying the expression of all genes in the control, 1-methyl-4-phenyl-1,2,3,6-tetrahydropyridine (MPTP) groups, and MPTP+SA groups. (C) Volcano plot displaying the differentially expressed genes (DEGs) of MPTP treatment compared with the control groups. (D) Volcano plot displaying the DEGs of SA treatment compared with the MPTP groups. (E) Kyoto Encyclopedia of Genes and Genomes (KEGG) enrichment revealed the pathways up-regulated by MPTP. (F) KEGG enrichment revealed the pathways down-regulated by SA. (G) Heatmaps displaying the expression of mitogen-activated protein kinase (MAPK) pathway genes in 3 groups. (H) Principal component analysis (PCA) of different protein changes in the MPTP vs. CTL and SA vs. MPTP groups. (I) Bubble diagram of KEGG pathway enrichment following SA treatment. (J) Protein changes in the Ephrin–Eph pathway after SA treatment in PD mice.

To further elucidate the pharmacological mechanism underlying the neuroprotective effects of SA in PD, multi-omics profiling was performed to characterize transcriptomic and proteomic alterations in the mouse STR (Fig. 7A). Claster analysis of whole-transcriptome profiles demonstrated distinct clustering patterns among the control, MPTP-challenged model, and SA-treated groups (Fig. 7B). Comparative analysis identified 1,297 differentially expressed genes between control and MPTP-challenged mice (Fig. 7C). Relative to the model group, SA treatment elicited 785 differentially expressed genes, including 434 down-regulated and 351 up-regulated genes (Fig. 7D). Kyoto Encyclopedia of Genes and Genomes (KEGG) enrichment analysis revealed that the MAPK signaling pathway was the most prominently up-regulated pathway upon MPTP challenge, whereas SA treatment led to its down-regulation (Fig. 7E and F). Gene set expression analysis further confirmed that SA treatment strongly suppressed the transcript levels of the MAPK pathway (Fig. 7G). The MAPK signaling pathway is critically implicated in the pathogenesis of PD, wherein its aberrant activation drives neuroinflammation and neuronal damage. Pharmacological suppression of the MAPK cascade has been shown to attenuate inflammatory responses and alleviate PD phenotypes. These results suggest that the suppression of the MAPK signaling pathway may play a pivotal role in the therapeutic effects of SA against PD.

Subsequent proteomic analysis revealed distinct proteome profiles across the 3 experimental groups (Fig. 7H and Fig. S31). In the MPTP model group, 246 proteins were significantly dysregulated (137 down-regulated and 109 up-regulated). SA treatment altered the expression of 329 proteins, with 147 down-regulated and 182 up-regulated (Fig. S32). KEGG enrichment analysis identified axon guidance as the most significantly modulated pathway following SA treatment (Fig. 7I), a finding further supported by Gene Ontology analysis (Fig. S33). Given the functional cross talk between axon guidance and MAPK signaling [37], key proteins in the axon guidance pathway were further evaluated. Most Ephrin and Eph family members were significantly up-regulated upon SA treatment, with EphrinB2 (Efnb2) exhibiting the highest fold change (Fig. 7J). This observation aligns with previous reports that glycosylation activates EphA receptor tyrosine kinase, thereby suppressing the MAPK pathway [38]. In summary, based on our multi-omics analysis, we hypothesize that SA inhibits OGA activity, leading to elevated *O*-GlcNAcylation levels in brain cells, which may stabilize the EphrinB2 protein and facilitate the activation of the Ephrin–Eph signaling pathway. This activated signaling is associated with the suppression of the MAPK pathway, potentially contributing to the amelioration of neuroinflammation.

### SA alleviates neuroinflammation via enhancing the *O*-GlcNAcylation of EphrinB2

**Fig. 8.**
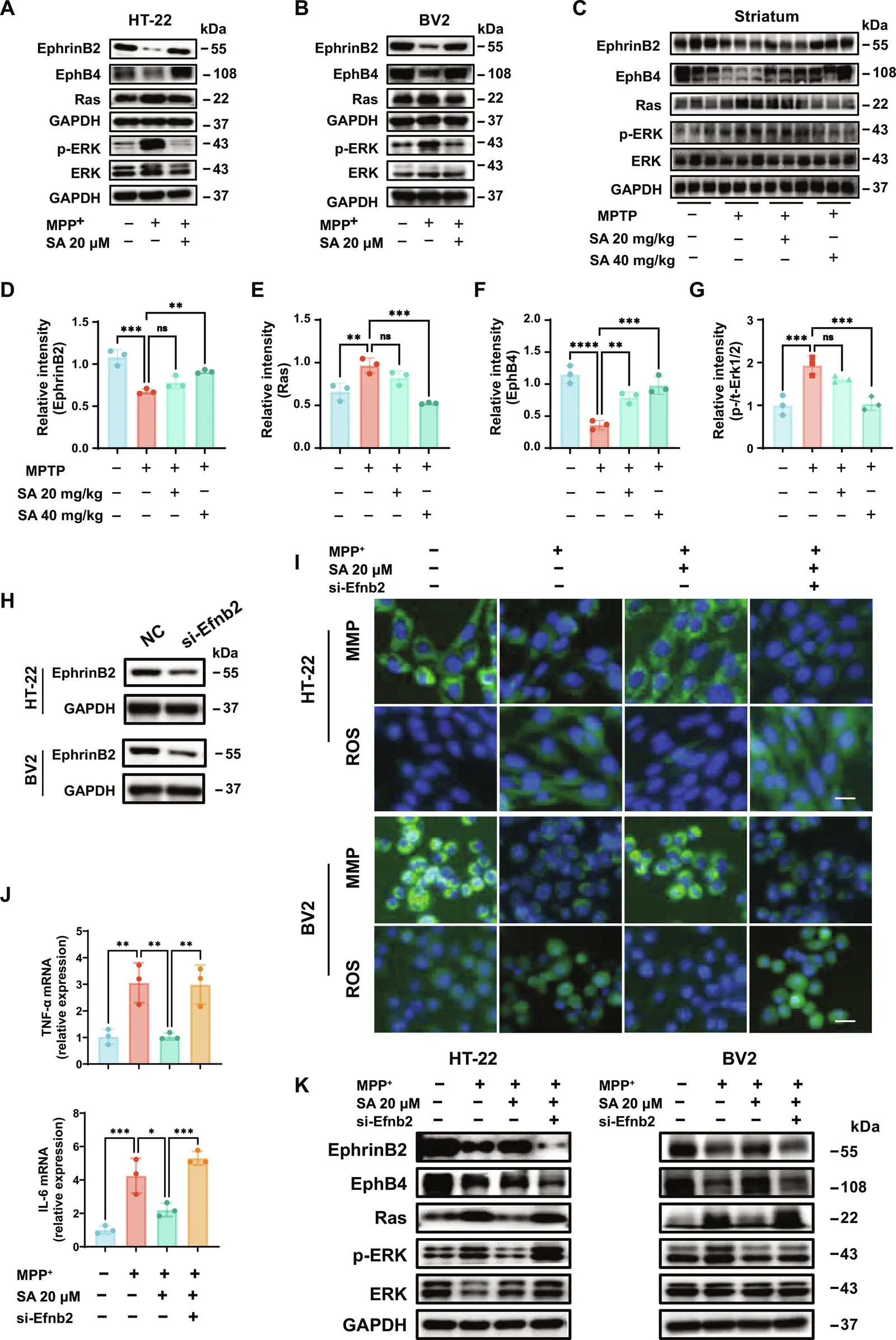
EphrinB2 mediates the neuroprotective effects of sennoside A (SA). Western blot images of EphrinB2, EphB4, Ras, p-ERK protein levels in 1-methyl-4-phenylpyridinium ion (MPP^+^)-challenged HT-22 cells (A) and BV2 cells (B). Western blot images (C) and quantification (D to G) of EphrinB2, EphB4, Ras, and p-ERK protein levels in mice brain. (H) Western blot images of HT-22 and BV2 cells transfected with si-Ctrl or si-Efnb2 messenger RNA (mRNA). (I) Fluorescence imaging of mitochondrial membrane potential (MMP) and reactive oxygen species (ROS) in transfected cells following MPP^+^ challenge with SA treatment; scale bar = 30 μm. (J) The mRNA expression of interleukin-6 (IL-6) and tumor necrosis factor-α (TNF-α) in transfected BV2 cells following MPP^+^ challenge with SA treatment. (K) The effects of Efnb2 knockdown on the expression of EphB4, Ras, and p-ERK in MPP^+^-challenged cells. All data are expressed as mean ± SD, *n* = 3; comparisons were analyzed by one-way analysis of variance with Dunnett’s tests. ns, *P* > 0.05; *P < 0.05; **P < 0.01; ***P < 0.005; ****P < 0.001.

Proteomic profiling pinpointed EphrinB2 as the top regulated protein within the axon guidance pathway upon SA treatment. To validate this finding, the expression levels of EphrinB2 and its receptor EphB4 in cellular PD models were first assessed. The results demonstrated that the expression levels of both EphrinB2 and EphB4 were significantly down-regulated in MPP^+^-challenged cells, while SA markedly restored their expression levels to near-normal levels (Fig. 8A and B). The MAPK pathway, particularly the Ras/MAPK signaling cascade, is closely associated with the pathogenesis of PD [39,40]. Notably, this pathway is negatively regulated by Ephrin–Eph signaling, whose activation suppresses the MAPK pathway and alleviates neuroinflammation [41]. Consistent with this, the results further demonstrated that SA treatment markedly attenuated the MPP^+^-induced up-regulation of Ras and phosphorylated ERK (p-ERK) in neuronal and microglial cells (Fig. 8A and B), and this effect was further corroborated in PD mouse models (Fig. 8C to G). To verify whether SA suppresses MAPK signaling and alleviates neuroinflammation through activating the Ephrin–Eph axis, *Efnb2* siRNA was transfected into HT-22 and BV2 cells. As shown in Fig. 8H to J, the protective effects of SA, including reduced ROS production, improved mitochondrial function, and suppressed inflammatory cytokine release, were largely abolished upon *Efnb2* knockdown. Moreover, the inhibitory influence of SA on the MAPK cascade was also completely abolished upon *Efnb2* silencing (Fig. 8K). Consistent with these observations, the neuroprotective effects of SA against PD was largely eliminated upon pharmacological blockade of the Ephrin–Eph axis using the specific inhibitor NVP-BHG712 (Fig. S34) [42]. These results further suggest that the therapeutic effect of SA is to improve neuroinflammation by activating the Ephrin–Eph signaling pathway and also provide a mechanistic basis for its anti-neuroinflammatory activity.

**Fig. 9.**
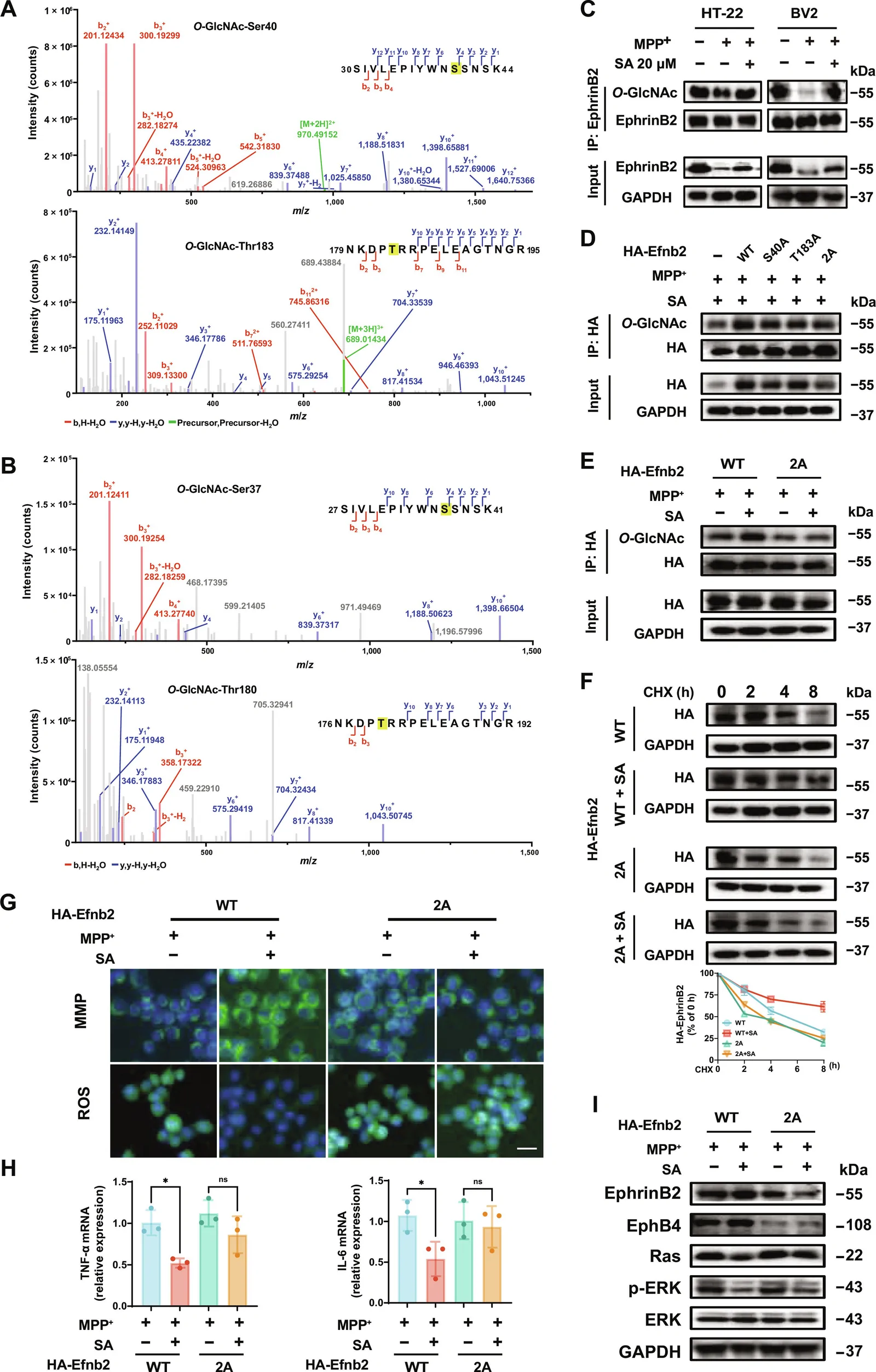
The *O*-GlcNAcylation of EphrinB2 is critical for the neuroprotective effects of sennoside A (SA). (A) The sites of mouse EphrinB2 *O*-GlcNAcylation were mapped by using mass spectrometry. (B) The sites of human EphrinB2 *O*-GlcNAcylation were mapped by using mass spectrometry. (C) The effects of SA on the *O*-GlcNAcylation of EphrinB2 in HT-22 and BV2 cells. (D and E) A co-immunoprecipitation (co-IP) assay was performed to validate the potential *O*-GlcNAcylation sites of EphrinB2 with anti-*O*-GlcNAc antibody. (F) Cycloheximide (CHX) chase assay measuring protein stability of WT-Efnb2-HA and S40A/T183A-Efnb2-HA with or without SA treatment. (G) Fluorescence imaging of mitochondrial membrane potential (MMP) and reactive oxygen species (ROS) in Efnb2-knockdown cells reconstituted with WT-Efnb2-HA or the S40A/T183A mutant, following 1-methyl-4-phenylpyridinium ion (MPP^+^) challenge and SA treatment; scale bar = 30 μm. (H) The messenger RNA (mRNA) expression of interleukin-6 (IL-6) and tumor necrosis factor-α (TNF-α) in Efnb2-knockdown cells reconstituted with WT-Efnb2-HA or the S40A/T183A mutant, following MPP^+^ challenge and SA treatment. (I) Effects of the S40A/T183A mutant on EphB4, Ras, and p-ERK protein levels in MPP^+^-challenged cells treated with or without SA. All data are expressed as mean ± SD, *n* = 3; comparisons were analyzed by one-way analysis of variance with Dunnett’s tests. ns, *P* > 0.05; *P < 0.05; **P < 0.01; ***P < 0.005; ****P < 0.001.

**Fig. 10.**
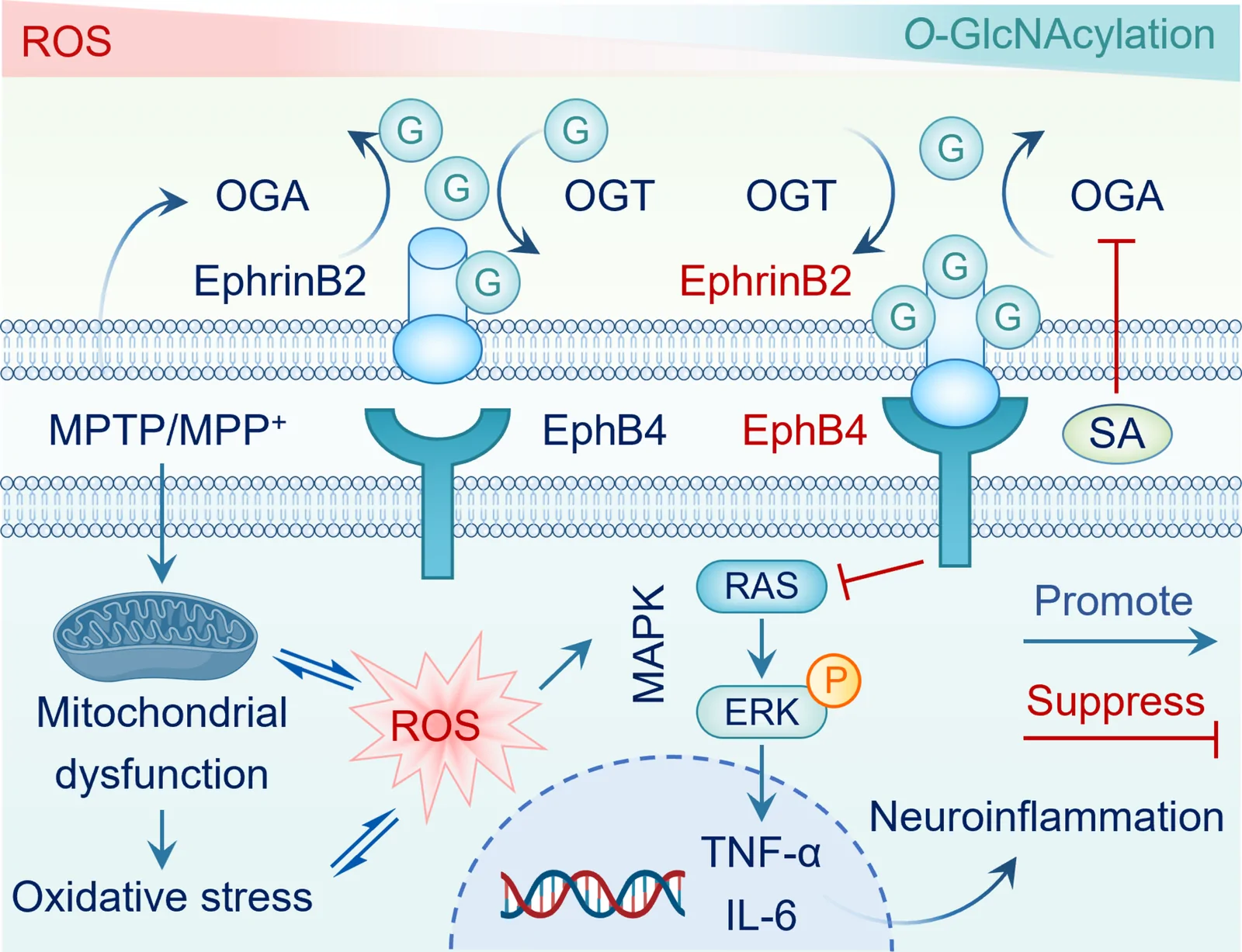
Schematic diagram illustrating the proposed mechanism by which sennoside A (SA) alleviates 1-methyl-4-phenyl-1,2,3,6-tetrahydropyridine (MPTP)-challenged neuroinflammation.

To explore the Ephrin–Eph activation mechanism, we first used liquid chromatography–tandem mass spectrometry (LC–MS/MS) to determine whether EphrinB2 undergoes *O*-GlcNAcylation. MS-based peptide profiling revealed that EphrinB2 is indeed *O*-GlcNAcylated and identified Ser40 and Thr183 as the major modification sites in mouse EphrinB2 (Fig. 9A), as well as Ser37 and Thr180 in human EphrinB2 under the same conditions (Fig. 9B). Co-immunoprecipitation (co-IP) confirmed the *O*-GlcNAcylation of EphrinB2 and showed that MPP^+^ challenge reduced this modification in PD cells, whereas SA treatment markedly elevated it (Fig. 9C). Rescue experiments in *Efnb2*-knockdown PD cells revealed that compared with wild-type (WT) reconstitution, the T183A single mutation moderately reduced *O*-GlcNAcylation, whereas the S40A single and S40A/T183A double mutations caused a drastic decrease (Fig. 9D). Consistent with these results, SA stimulation markedly enhanced the *O*-GlcNAcylation of WT EphrinB2 but failed to affect the double mutant (Fig. 9E). Moreover, SA prolonged the half-life of WT EphrinB2 but had no effect on the S40A/T183A mutant, indicating that SA-mediated *O*-GlcNAcylation at these sites stabilizes EphrinB2 (Fig. 9F). Subsequent phenotypic assays clearly demonstrated that the neuroprotective effects of SA were fully rescued by the overexpression of WT EphrinB2. In contrast, reconstitution with mutated EphrinB2 failed to recapitulate the protective effects of SA, leaving cellular PD pathological phenotypes largely unaltered (Fig. 9G and H), thereby underscoring the essential role of Ser40/Thr180 *O*-GlcNAcylation in EphrinB2-mediated SA efficacy against PD. These phenotypic observations were further corroborated by WB analysis of MAPK-pathway-related proteins. In *Efnb2*-knockdown PD cells, WT EphrinB2 reconstitution enabled SA to markedly attenuate MAPK activation. However, this effect was entirely nullified by the S40A/T183A mutation, revealing that the MAPK-suppressive activity of SA is critically dependent on EphrinB2 *O*-GlcNAcylation at Ser40 and Thr183 (Fig. 9I). Collectively, these findings demonstrate that SA substantially elevates EphrinB2 *O*-GlcNAcylation, thereby activating the Ephrin–Eph axis and suppressing MAPK signaling, which in turn alleviates MPTP-induced neuroinflammation and PD-related phenotypes (Fig. 10).

## Discussion

PD is a prevalent progressive neurodegenerative disorder characterized by dopaminergic neuron loss and Lewy body formation, with multifactorial pathogenesis involving oxidative stress, neuroinflammation, and mitochondrial dysfunction [43]. Dysregulation of protein glycosylation, particularly *O*-GlcNAcylation, is increasingly recognized as a key player in neurodegenerative diseases like PD; the restoration of this modification on key proteins offers a potential therapeutic strategy. As the sole enzyme responsible for *O*-GlcNAc removal, OGA represents an attractive therapeutic target, the pharmacological inhibition of which provides a direct means to elevate *O*-GlcNAc levels and counteract neurodegeneration. However, no safe and potent OGA inhibitor has yet advanced to clinical development for PD, underscoring an urgent need for selective, well-tolerated OGA-targeted therapeutics. To address this gap, we designed a novel, specific OGA fluorescent substrate addressing existing detection limitations and established an HTS platform, identifying SA from an in-house phytochemical library as a potent OGA inhibitor (Figs. 2 and 3). This screening strategy aligns with the research focus on natural products as a valuable resource for PD drug development, addressing the current shortcomings of natural product research (e.g., unclear molecular targets and lack of systematic mechanism). SA exhibited high metabolic stability and effectively inhibited OGA activity across multiple assay levels. Critically, in vitro (MPP^+^/6-OHDA-challenged cell models) and in vivo (MPTP-challenged mouse models) studies demonstrated that SA administration markedly mitigated PD-related pathological damages, including behavioral deficits, dopaminergic neuron apoptosis, neuroinflammation, oxidative stress, and decreased *O*-GlcNAcylation levels (Figs. 4 to 6), providing direct evidence supporting OGA inhibition as a viable strategy for PD intervention.

To elucidate the pharmacological mechanism underlying the neuroprotective effects of SA against PD, we performed multi-omics analyses and identified the MAPK pathway as a critical mediator of SA’s therapeutic action (Fig. 7). The MAPK pathway, particularly the p38 MAPK and ERK signaling cascades, is closely associated with the pathogenesis of PD [44–46]. Activation of the MAPK pathway has been implicated in various pathological processes in PD, including neuroinflammation, oxidative stress, and mitochondrial dysfunction [45–47]. Pharmacological inhibition of this pathway has been shown to alleviate inflammation and improve PD-like phenotypes in preclinical models [48]. Previous work has demonstrated that *O*-linked β-*N*-acetylglucosamine transferase (OGT) can bind to p38, enhancing its *O*-GlcNAcylation and suppressing its phosphorylation, thereby attenuating MAPK pathway activation [49]. However, the connection between OGA, *O*-GlcNAc dynamics, and MAPK signaling in PD remained unexplored. Our study bridges this gap by providing compelling evidence that OGA inhibition with SA attenuates MAPK activation. Mechanistically, SA directly inhibits OGA in neuronal cells, which elevates *O*-GlcNAcylation and further blunts MAPK signaling activation, thereby mitigating neurotoxic inflammatory cascades. Notably, such cytoprotective actions are not restricted to neurons; SA also potently represses microglial inflammatory overactivation, as validated by diminished secretion of pro-inflammatory cytokines following SA intervention (Figs. 6 to 8). Together, these findings demonstrate that SA exerts neuroprotection through OGA-mediated MAPK suppression, alongside a direct inhibitory effect on microglial inflammation. Accordingly, this study establishes a distinct mechanistic framework wherein SA-mediated OGA inhibition attenuates MAPK-mediated neuroinflammation via coordinate regulatory actions on neuronal and microglial compartments.

Proteomic analysis revealed, for the first time, that axon guidance represents the most profoundly altered pathway following OGA inhibition, characterized by marked up-regulation of key guidance molecules including Ephrin and EphA proteins (Fig. 7). This systematic discovery provides a novel perspective for understanding how OGA inhibition exerts neuroprotective effects through the regulation of receptor tyrosine kinase signaling networks, which was not previously reported in PD research. Of particular significance, we identified and validated that *O*-GlcNAcylation, a PTM functionally analogous to *N*-glycosylation [50], could enhance EphrinB2 protein stability, thereby activating the EphrinB2–EphB4 signaling pathway and subsequently suppressing the MAPK signaling cascade (Figs. 8 and 9). EphrinB2 has been primarily implicated in embryonic cardiogenesis and angiogenesis, with previous research largely restricting its functions to the vascular system [51]. Herein, we reveal for the first time its therapeutic effect against PD and broaden the functional scope of EphrinB2. Notably, this finding fills a critical gap in the field by establishing that EphrinB2 *O*-GlcNAcylation plays a pivotal role in PD pathogenesis and that pharmacological OGA inhibition restores its modification status to confer therapeutic benefit. Unlike classical OGA inhibitors such as T-G and MK-8719, which primarily exert their effects through a global and nonselective elevation of *O*-GlcNAcylation, SA acts through a fundamentally distinct mechanism, specifically the restoration of EphrinB2 *O*-GlcNAcylation and subsequent activation of the EphrinB2–EphB4 signaling axis. This targeted pathway is intrinsically linked to synaptic maintenance and plasticity. In contrast, the synaptic toxicity associated with broad-spectrum OGA inhibitors, such as MK-8719, has constrained their clinical development, including ongoing phase II trials for Alzheimer’s disease. Consequently, by engaging the Ephrin–Eph pathway to support synaptic function while circumventing the off-target synaptic toxicity of classical OGA inhibitors, SA provides a mechanistically distinct and potentially safer therapeutic strategy for PD. Nonetheless, several limitations of this study warrant consideration. First, this work lacks in vivo validation through *Efnb2* knockout or knockdown at the animal level.

Establishing a causal role for Ephrin signaling in the observed effects of OGA inhibition will require future studies employing conditional EphrinB2-deficient mouse models. Second, although the MPTP-induced PD model is a well-established acute paradigm for studying dopaminergic neurodegeneration, it does not fully recapitulate the chronic and progressive nature of human PD. Therefore, complementary validation in genetic models that more faithfully mirror the spontaneous onset and gradual progression of the disease, such as α-synuclein transgenic mice, will be critical for establishing the translational relevance of our findings.

## Conclusion

In summary, this work identifies a naturally occurring OGA inhibitor that exerts anti-PD effects by promoting EphrinB2 *O*-GlcNAcylation via Ser40/Thr183 and suppressing the MAPK pathway. First, a specific fluorogenic substrate for OGA was developed through virtual screening combined with in vitro biochemical validation, enabling the construction of a fluorescence-based HTS assay suitable for complex biological systems. Following HTS of an in-house phytochemical library, SA was identified as a potent competitive OGA inhibitor. Subsequent characterization revealed that SA exhibits favorable BBB permeability, a well-tolerated safety profile, and robust inhibitory activity both in vitro and in vivo. Pharmacological inhibition of OGA by SA markedly restores *O*-GlcNAcylation levels and confers neuroprotection against PD. More importantly, EphrinB2 was identified as a key protein in both neuronal and glial cells, whose *O*-GlcNAcylation is enhanced upon OGA inhibition, thereby activating the EphrinB2–EphB4 signaling pathway and attenuating MAPK-driven neuroinflammation. Notably, SA also directly suppresses glial inflammatory responses, suggesting that its neuroprotective effects involve both OGA-dependent modulation in neurons and direct anti-inflammatory actions on glial cells. Collectively, this work uncovers a mechanistic cascade in which OGA inhibition promotes EphrinB2 *O*-GlcNAcylation to counteract MAPK-mediated neuroinflammation, providing both a candidate anti-PD agent and a novel mechanistic framework for PD intervention.

## Materials and Methods

### Materials and reagents

Recombinant enzymes including mouse EphrinB2 (Cat. HY-P73017), human EphrinB2 (Cat. HY-P77645), dipeptidyl peptidase 9 (DPP9; Cat. HY-P703641), PREP (Cat. HY-P73692), human OGA (from *Bacteroides thetaiotaomicron*, Cat. HY-P79242, purity >95%), and human hyaluronidase (Cat. HY-108903) were purchased from MCE (Shanghai, China). β-*N*-Acetylhexosaminidase (from *Streptomyces plicatus*) was obtained from New England Biolabs (Cat. P0721S, purity >95%). Bovine serum albumin, acetylcholinesterase (Cat. C3389, derived from *Electrophorus electricus*), butyrylcholinesterase (sourced from human plasma, Cat. B4186), human CES1 (Cat. E0287), human CES2 (Cat. E0412), bovine testicular hyaluronidase (from bovine testes, Cat. H3506), α-glucosidase (from *Saccharomyces cerevisiae*, Cat. G5003), and β-glucosidase (from almonds, Cat. 49290) were all obtained from Sigma-Aldrich (St. Louis, MO, USA). T-G (Cat. HY-12588), MPP^+^ (Cat. HY-W008719), cycloheximide (CHX; HY-12320), and NVP-BHG712 (Cat. SML0333) were all purchased from MCE (Shanghai, China). Mouse IL-6 (Cat. 1210602), IL-10 (Cat. 1211002), and TNF-α (Cat. 1217202) ELISA kits were all obtained from Dakewe Biotech (China). Plasmids (pCMV-WT-*Efnb2*-HA, pCMV-WT-S40A-HA, pCMV-WT-T183A-HA, and pCMV-WT-2A-HA) were synthesized by Suzhou Junji Gene Technology Co., Ltd. Protein A+G agarose was purchased from Beyotime (Shanghai, China). All chemicals were commercial products of analytical grade.

### Molecular docking simulations

To identify potential fluorescent substrates targeting OGA, molecular docking simulations were carried out by means of AutoDock Vina (v1.1.2). The crystal structures of OGA (PDB IDs: 5TKE, 5M7U, 5VVU, 5VVT, and 5VVO) were retrieved from the PDB and prepared by removing crystallographic water and nonessential ligands, adding hydrogen atoms, and assigning Kollman united-atom charges. Fluorophores were selected based on OGA substrate preference, and their 3-dimensional (3D) structures were energy-minimized with ChemBio3D Ultra 14.0. A docking grid of 70 × 70 × 70 Å^3^ (spacing 0.375 Å) was centered on the catalytic pocket. The catalytic conformation was defined as the distance between the *N*-acetylglucosamine carbonyl carbon and the OGA active-site residue ASN-175. Protein–ligand interactions were visualized using PyMOL (v2.4) and Discovery Studio (2020). Virtual screening of naphthalimide derivatives was then conducted under default parameters using the OGA structure (PDB ID: 5VVO). The compound with the lowest calculated binding energy was selected for synthesis and further experimental validation.

### Synthesis procedure and structural characterization of OGA probe candidates

#### The synthesis of compound NCO

4-Bromo-1,8-naphthalic anhydride (2 g, 7.2 mmol) was dissolved in anhydrous ethanol, and then *n*-butylamine (1,069 ml, 10.8 mmol) was added and reacted at 80 °C for 6 h. Solid precipitation can be observed. After the reaction was completed, it was cooled to room temperature, then concentrated, filtered, separated by column chromatography, and dried to obtain 1.96 g of yellow powder (NB; yield: 82%). ^1^H NMR (400 MHz, CDCl_3_) *δ* 8.65 (dd, *J* = 7.3, 1.1 Hz, 1H), 8.56 (dd, *J* = 8.5, 1.1 Hz, 1H), 8.41 (d, *J* = 7.8 Hz, 1H), 8.04 (d, *J* = 7.9 Hz, 1H), 7.84 (dd, *J* = 8.5, 7.3 Hz, 1H), 4.21 to 4.13 (m, 2H), 1.77 to 1.65 (m, 2H), 1.51 to 1.38 (m, 2H), 0.98 (t, *J* = 7.4 Hz, 3H). ^13^C NMR (101 MHz, CDCl_3_) *δ* 163.5, 133.0, 131.8, 131.0, 130.9, 130.5, 130.0, 128.8, 127.9, 123.0, 122.1, 40.2, 30.0, 20.2, 13.7. HRMS (ESI, *m*/*z*): [M + H]^+^: calculated for C_16_H_14_BrNO_2_H^+^ 332.0208, found 332.0277.

**Fig. 11.**
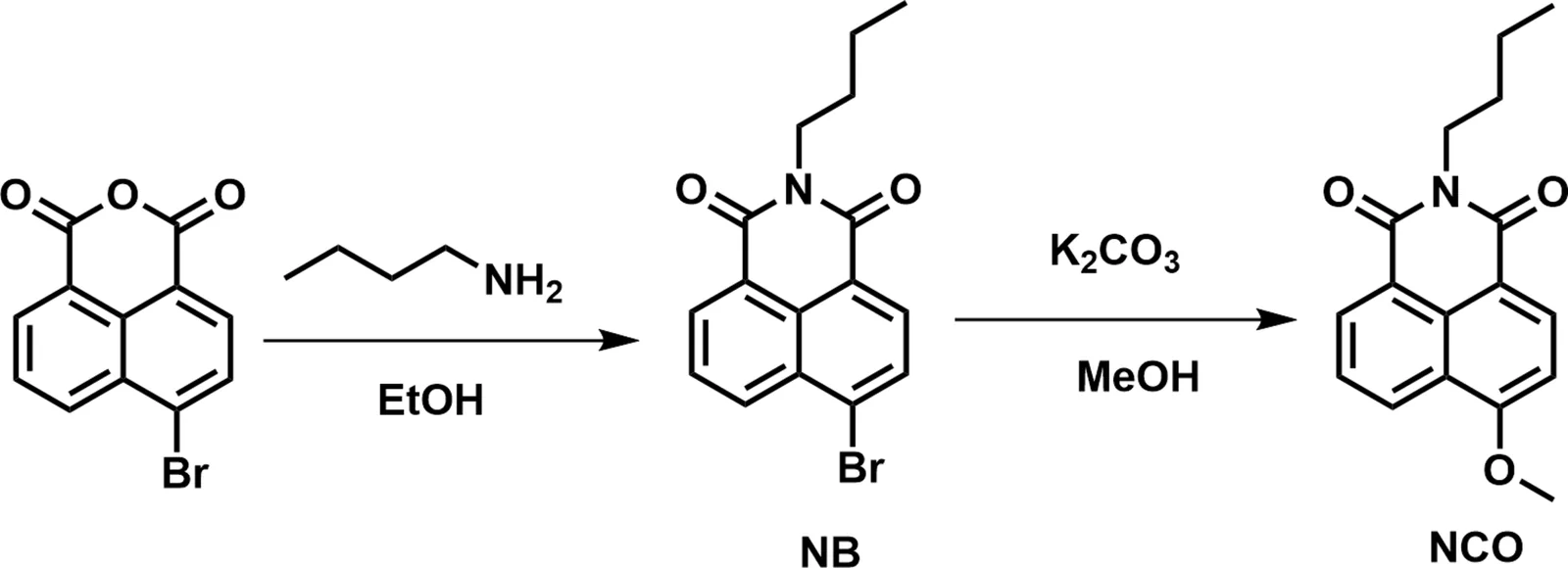
Synthesis route of NCO.

Compound NB (1 g, 3 mmol) was dissolved in 50 ml of methanol. Then, potassium carbonate (622 mg, 4.5 mmol) was added. The reaction mixture was stirred at 80 °C for 8 h. After the reaction was completed, it was cooled in an ice-water bath, resulting in the precipitation of a large amount of yellow solid. The solid was filtered, washed with a large amount of water, and then dried under vacuum to obtain a yellow solid weighing 792.4 mg (NCO; yield: 93%) (Fig. 11). ^1^H NMR (400 MHz, CDCl_3_) *δ* 8.60 (dd, *J* = 7.3, 1.3 Hz, 1H), 8.57 to 8.53 (m, 2H), 7.70 (dd, *J* = 8.4, 7.3 Hz, 1H), 7.04 (d, *J* = 8.3 Hz, 1H), 4.19 to 4.14 (m, 2H), 4.13 (s, 3H), 1.77 to 1.65 (m, 2H), 1.52 to 1.38 (m, 2H), 0.97 (t, *J* = 7.4 Hz, 3H). ^13^C NMR (101 MHz, CDCl_3_) *δ* 165.0, 164.5, 161.2, 133.9, 132.0, 129.8, 129.0, 126.4, 124.0, 123.0, 115.6, 105.6, 56.7, 40.6, 30.7, 20.9, 14.3. HRMS (ESI, *m*/*z*): [M + H]^+^: calculated for C_17_H_17_NO_3_H^+^ 284.1208, found 284.1287. The purity is 85.33% (Fig. S13A).

#### The synthesis of compound NH

**Fig. 12.**
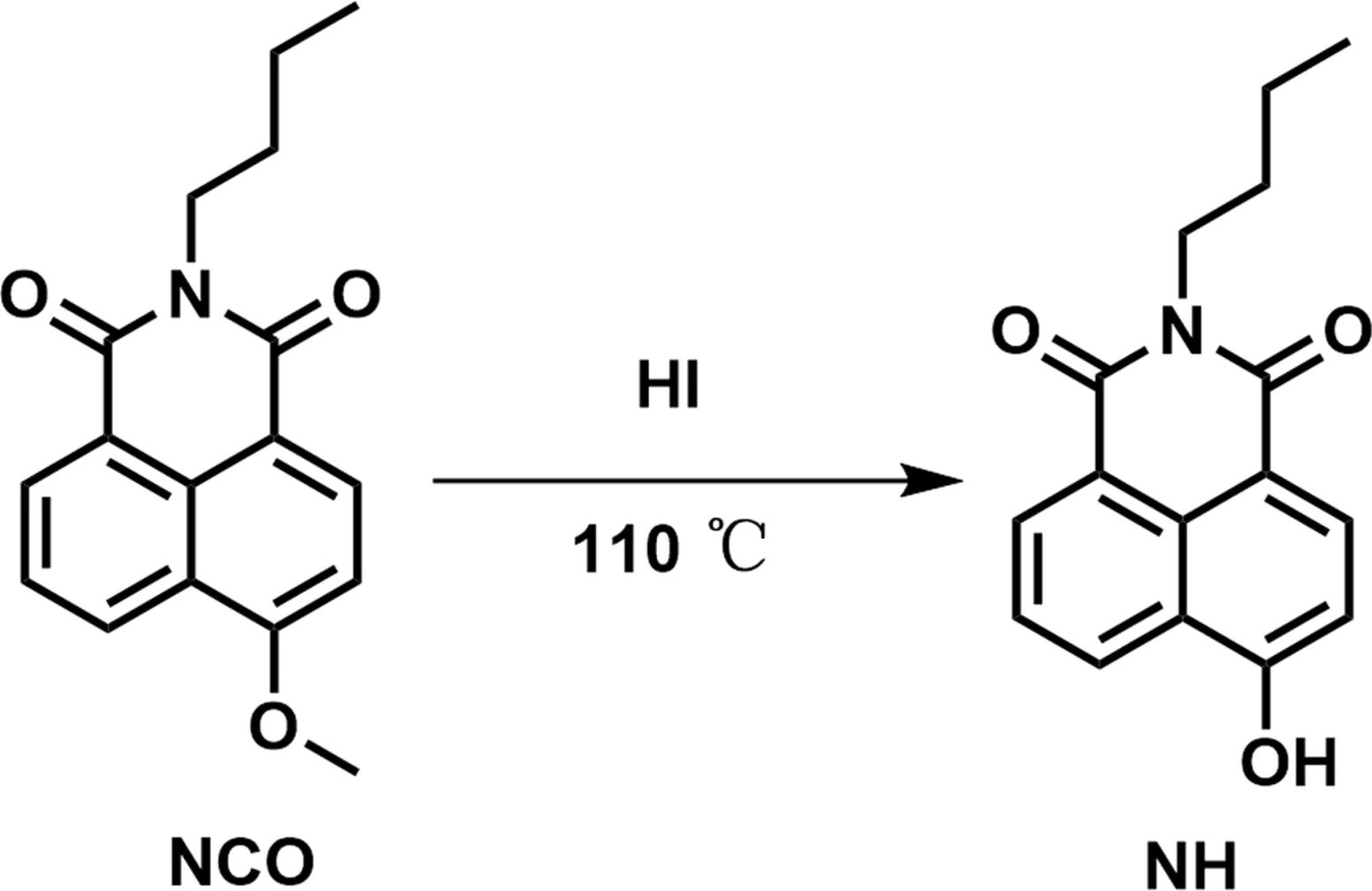
Synthesis route of NH.

Compound NCO (1.0 g, 3.53 mmol) was reacted in concentrated HI (excess) at 110 °C for 6 h. The reaction mixture was first cooled, then diluted with water, and extracted with dichloromethane. The combined organic layers were washed with brine, dried over anhydrous Na_2_SO_4_, and concentrated in vacuo. The crude product was then recrystallized from ethyl acetate and petroleum ether to afford NH as a yellow solid (NH, 906 mg, yield: 95%) (Fig. 12). ^1^H NMR (600 MHz, dimethyl sulfoxide-d_6_ [DMSO-d_6_]) *δ* 11.81 (s, 1H), 8.50 (d, *J* = 8.2 Hz, 1H), 8.43 (d, *J* = 7.1 Hz, 1H), 8.32 (d, *J* = 8.2 Hz, 1H), 7.73 (t, *J* = 7.8 Hz, 1H), 7.13 (d, *J* = 8.2 Hz, 1H), 4.00 (m, 2H), 1.58 (m, 2H), 1.33 (dd, *J* = 14.8, 7.4 Hz, 2H), 0.91 (t, *J* = 7.3 Hz, 3H). ^13^C NMR (101 MHz, DMSO-d_6_) *δ* 163.6, 162.9, 160.2, 133.5, 131.0, 129.1, 128.8, 125.5, 122.3, 121.8, 112.6, 109.9, 29.7, 19.8, 13.7. HRMS (ESI, *m*/*z*): [M + H]^+^: calculated for C_16_H_15_NO_3_H^+^ 270.1052, found 270.1132. The purity is 97.98% (Fig. S13B).

#### The synthesis of compound NAGN

**Fig. 13.**
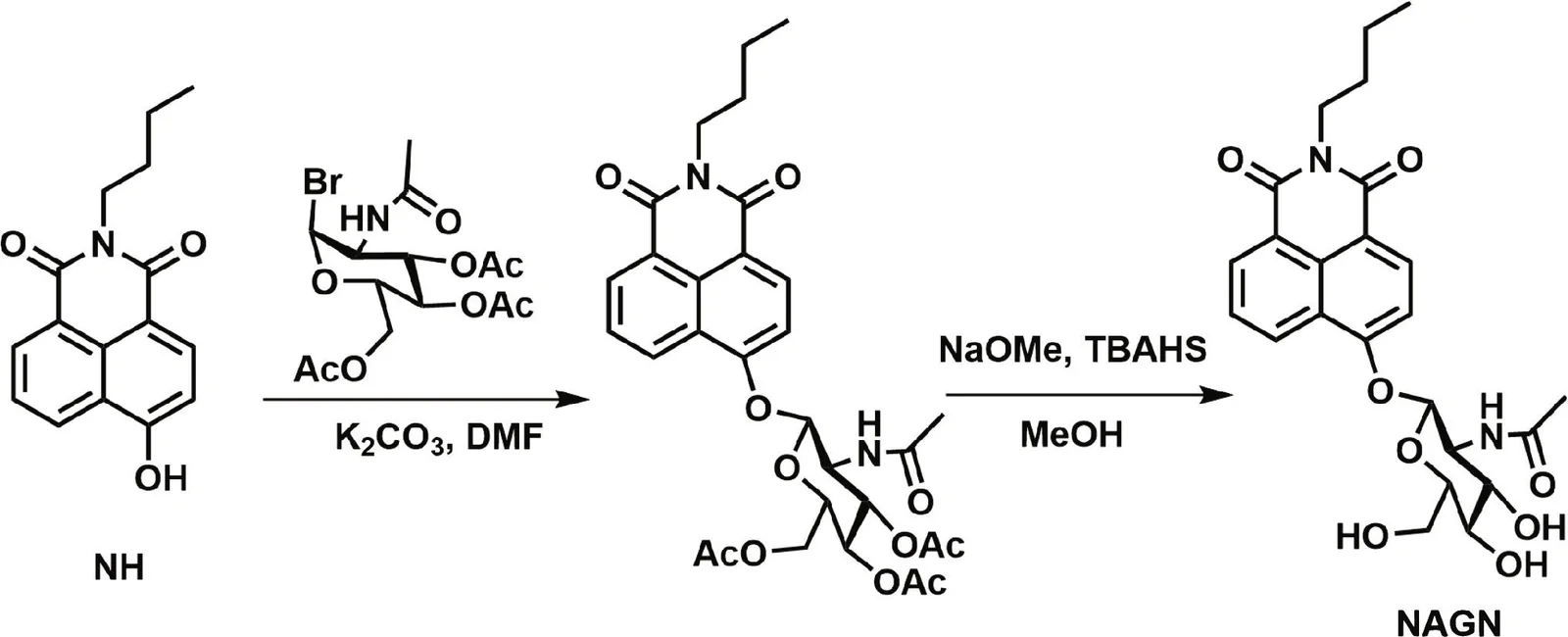
Synthesis route of NAGN.

Compound NH (300 mg, 1.11 mmol) and 2-acetylamino-3,4,6-triacetyl-2-deoxy-α-d-glucopyranosyl bromide (200 mg, 0.49 mmol) were dissolved in dimethylformamide. Potassium carbonate (165 mg, 1.2 mmol) was added, and the reaction mixture was stirred at 80 °C for 12 h. The reaction mixture was cooled down to the ambient temperature, diluted using water, and thereafter underwent extraction with dichloromethane. The integrated organic layers were concentrated in vacuo to obtain a crude product. To this crude product, sodium methoxide (162 mg, 3 mmol) and tetrabutylammonium hydrogen sulfate (167 mg, 0.5 mmol) were added, and the mixture was stirred in methanol for 6 h. The product obtained was purified through column chromatography, giving rise to NAGN in the form of a white solid (NAGN, 85 mg; yield: 37%) (Fig. 13). ^1^H NMR (400 MHz, DMSO-d_6_) *δ* 8.48 (dd, *J* = 7.8, 4.6 Hz, 2H), 8.43 (d, *J* = 8.3 Hz, 1H), 7.93 (d, *J* = 9.1 Hz, 1H), 7.83 (t, *J* = 7.8 Hz, 1H), 7.46 (d, *J* = 8.3 Hz, 1H), 5.25 to 5.17 (m, 3H), 4.70 (t, *J* = 5.8 Hz, 1H), 4.04 (td, *J* = 8.0, 7.5, 4.7 Hz, 3H), 3.79 (d, *J* = 4.1 Hz, 1H), 3.61 to 3.51 (m, 3H), 1.83 (s, 3H), 1.63 to 1.57 (m, 2H), 1.36 (h, *J* = 7.4 Hz, 2H), 0.94 (t, *J* = 7.3 Hz, 3H). ^13^C NMR (101 MHz, DMSO-d_6_) *δ* 169.8, 163.5, 162.8, 158.2, 132.8, 131.1, 128.5, 128.2, 126.6, 123.0, 121.9, 115.5, 109.6, 99.8, 77.5, 73.5, 70.1, 60.6, 55.1, 29.7, 23.1, 19.8, 13.7. HRMS (ESI, *m*/*z*): [M + H]^+^: calculated for C_24_H_28_N_2_O_8_H^+^ 473.1846, found 473.1925. The purity is 96.56% (Fig. S13C).

### General procedure for monitoring OGA activity

OGA activity was assayed in a 0.1-ml system containing 100 mM phosphate-buffered saline (PBS; pH 7.4), enzyme, and probe (NAGN). The reaction was initiated by adding NAGN, incubated at 37 °C for 30 min in a shaking water bath, and then terminated with an equal volume of ice-cold acetonitrile. Fluorescence was measured using a SpectraMax iD3 microplate reader (Molecular Devices) at 450/550 nm. Alternatively, samples were centrifuged at 20,000 × g for 30 min and analyzed by HPLC–UV.

### Enzymatic hydrolysis of 4MU-GlcNAc and NAGN by OGA versus NAG

To compare the specificity of NAGN and the commercial probe 4MU-GlcNAc toward OGA and NAG enzymes, all assays were performed in 100 mM PBS (pH 7.4). Reactions (0.1 ml total volume) containing PBS and an equal concentration of the respective enzyme (0.1 μg/ml) were gently mixed. The reaction was initiated by adding the substrate at an identical concentration (50 μM). Fluorescence intensity was continuously monitored at 37 °C for 60 min using a microplate reader (SpectraMax iD3, Molecular Devices, San Jose, USA) with excitation/emission wavelengths set at 365/445 nm for 4MU and 450/550 nm for NH.

### HPLC–UV analysis of both NAGN and its hydrolytic product NH

Chromatographic separation of NAGN and its metabolite NH was achieved using an HPLC system (Shimadzu, Japan) comprising a CBM-20A system controller, an LC-20AD binary pump, an SPD-M20A UV detector, an SIL-20AC autosampler, and a Hypersil ODS C8 column (2.1 × 50 mm, 5 μm; Shimadzu). The mobile phase consisted of (A) 0.1% aqueous formic acid and (B) methanol. A gradient elution program was employed as follows: 0 to 0.01 min, 30% B; 0.01 to 0.5 min, 30% to 70% B; 0.5 to 4 min, 70% to 90% B; 4 to 5 min, 90% B; 5 to 5.05 min, 90% to 30% B; and 5.05 to 7.5 min, 30% B. The total run time was 7.51 min, with a constant flow rate of 0.4 ml/min, and the column temperature was maintained at 40 °C. The purity of compounds was measured using a C18 column (2.1 × 150 mm, 5 μm; Shimadzu), and other conditions were the same as above.

### Selectivity of NAGN toward OGA

To determinate the selectivity of NAGN in detecting OGA, a series of hydrolases (OGA, DPP9, PREP, CES1, CES2, acetylcholinesterase, butyrylcholinesterase, bovine testicular hyaluronidase, α-glucosidase, hyaluronidase, β-glucosidase, and NAG) and bovine serum albumin (each at a concentration of 1 μg/ml) were individually incubated with the probe NAGN (10 μM, DMSO < 1%) at 37 °C for 30 min, respectively. The reaction was terminated with an equal volume of ice acetonitrile and then measured with a microplate reader (SpectraMax iD3) at excitation/emission wavelengths of 450/550 nm.

### Enzymatic kinetic analyses

To determine the kinetic parameters of NAGN metabolism by recombinant human OGA and in HLC, incubation conditions were first optimized to ensure the linearity of metabolite formation with respect to both incubation time and protein concentration. The reaction mixtures containing HLC (0.1 mg/ml) or OGA (0.2 μg/ml) in PBS were preincubated at 37 °C for 3 min, then were initiated by adding NAGN at gradient concentrations (0 to 300 μM for HLC and 0 to 500 μM for OGA), and incubated for 40 and 10 min, respectively; next, they were terminated with an equal volume of ice-cold acetonitrile. Sample fluorescence was detected via a SpectraMax iD3 microplate reader (Molecular Devices, San Jose, USA) at 450-nm excitation and 550-nm emission or quantified by HPLC–UV. The Michaelis–Menten equation (Eq. (1)) was fitted to the initial velocity data using GraphPad Prism 10.0 (GraphPad Software, San Diego, CA, USA) to obtain the kinetic parameters *V*_max_ and *K*_m_. The results were visualized using Eadie–Hofstee plots. The intrinsic clearance (*CL*_int_) was calculated as the ratio of *V*_max_ to *K*_m_.

$$V=\frac{V_{\max}\;\times \;\left[S\right]}{K_{m}\;+\;\left[S\right]}$$ (1)

where *V* is the metabolic rate, *V*_max_ is the maximum reaction velocity, *K*_m_ is the Michaelis constant, and [*S*] is the substrate concentration.

### HTS of OGA inhibitors

A NAGN-based screening platform was established to identify potential OGA inhibitors from natural products. The enzymatic reaction was performed in a 100-μl mixture containing PBS (100 mM, pH 7.4), OGA (0.2 μg/ml), and each test compound (20 μM). Following preincubation at 37 °C for 3 min, the reaction was initiated by adding the fluorescent substrate NAGN (50 μM) and allowed to proceed at 37 °C for 30 min. The reaction was quenched with 100 μl of ice-cold acetonitrile, and fluorescence was measured using a SpectraMax iD3 microplate reader (Molecular Devices, Austria) at excitation/emission wavelengths of 450/550 nm. OGA residual activity (%) was calculated as (fluorescence with inhibitor/fluorescence of DMSO control) × 100%, and results were plotted using Origin 2021.

### Inhibitory mechanism of SA

The inhibitor’s half-maximal inhibitory concentration (IC_50_) was first determined using a series of concentration gradients. Its specificity toward OGA was assessed by comparing its effect on OGA versus NAG activity. To evaluate TDI, SA was preincubated with OGA for 3 or 33 min before the reaction started. The IC_50_ values obtained at these different preincubation times were compared; a significant increase in IC_50_ with longer preincubation suggests TDI.

To elucidate the inhibition mechanism, a series of reactions were performed in a 100-μl PBS-based system containing the substrate NAGN (20, 40, 60, and 80 μM) and SA at various final concentrations (0, 0.05, 0.2, 0.4, and 0.8 μM). The mode of inhibition was determined by analyzing the intersection patterns in the Lineweaver–Burk plot. The inhibition constant (*K*_i_) was subsequently calculated by globally fitting the initial velocity data to the appropriate model for competitive (Eq. (2)), noncompetitive (Eq. (3)), or mixed (Eq. (4)) inhibition.

$$V=\frac{V_{\max}\;\times \;S}{K_{m}\left(1+I/K_{i}\right)+S}$$ (2)

$$V=\frac{V_{\max}\;\times \;S}{\left(K_{m}+S\right)\times \left(1+I/K_{i}\right)}$$ (3)

$$V=\frac{V_{\max}\;\times \;S}{\left(K_{m}+S\right)\times \left(1+I/\alpha K_{i}\right)}$$ (4)

where *V* is the initial velocity of NAGN hydrolysis by OGA, *V*_max_ is the maximum reaction velocity, *S* is the substrate (NAGN) concentration, *I* is the inhibitor (SA) concentration, *K*_m_ is the Michaelis constant for NAGN, and *K*_i_ is the inhibition constant.

### Inhibition profile of SA against other enzymes

The inhibitory activity of SA (20 μM) against various hydrolases was assessed using their respective optical substrates under the following conditions: for CES1, human liver microsomes (HLMs) 0.5 μg/ml, *N*-nitroso-L-methionine ethyl ester 20 μM, pH 6.5, bioluminescence; for CES2, HLMs 1 μg/ml, fluorescein diacetate 20 μM, pH 7.4, *λ*_ex/em_ 480/525 nm; for human pancreatic lipase, purified enzyme 10 μg/ml, DDAO-ol 10 μM, Tris–HCl pH 7.4, *λ*_ex/em_ 600/660 nm; for PREP, mouse brain S9, ZGPB-DDAO 2 μM, pH 7.4, *λ*_ex/em_ 600/650 nm; and for NAG, 4MU-GlcNAc 2 μM, pH 7.4, *λ*_ex/em_ 365/445 nm. Reactions were terminated with LDR (CES1) or acetonitrile (CES2, PL, PREP, and NAG) as appropriate.

### Molecular docking and MD simulation

To elucidate the inhibitory mechanism of SA against OGA, a molecular docking simulation was performed using AutoDock Vina (version 1.1.2). The crystal structure of OGA (PDB ID: 5VVO) was retrieved from the PDB. Prior to docking preparation, redundant crystal water molecules and nonessential ligands were removed, followed by hydrogen atom addition and assignment of Gasteiger–Marsili united-atom charges. The 3D structure of SA was constructed and subjected to energy minimization using ChemBio3D Ultra 14.0. A docking box with dimensions of 70 × 70 × 70 Å^3^ and a grid spacing of 0.375 Å was centered on the catalytic pocket of OGA for molecular docking calculation. The optimal conformation with the lowest binding energy was selected for subsequent visual analysis.

The output conformation with the highest binding affinity generated by molecular docking is used as the initial file for MD simulations. Each OGA–ligand complex was solvated in a dodecahedral TIP3P water box and neutralized with 0.15 M NaCl to achieve a physiological ionic strength of approximately 150 mM. The system underwent 50,000 steps of steepest-descent energy minimization, with a maximum force (*E*_max_) threshold of 1,000 kJ mol^−1^ nm^−1^. Equilibration was performed in 2 stages: first, 100 ps of NVT (constant number of particles, volume, and temperature) heating from 0 to 310 K with position restraints (*k* = 1,000 kJ mol^−1^ nm^−2^) applied to all heavy solute atoms, followed by 100 ps of NPT (constant number of particles, pressure, and temperature) relaxation at 1 bar, with identical restraints maintained. The production MD simulations were executed for 100 ns under periodic boundary conditions at 310 K and 1 bar, employing a V-rescale thermostat and the Parrinello–Rahman barostat.

### Cell culture and imaging

SH-SY5Y and related cell lines were cultured under standard conditions. After reaching 80% confluence in 6-well plates, cells were assigned to the control, model, and inhibitor groups, receiving the base medium with DMSO, MPP^+^/6-OHDA alone, or SA and MPP^+^/6-OHDA, respectively, for 24 h.

### Cytotoxicity assays

The CTC, BEAS-2B, A549, HT-22, and SH-SY5Y cell lines, purchased from the Chinese Academy of Sciences Cell Bank, were cultured in a high-glucose Dulbecco’s modified Eagle medium (DMEM; HyClone, USA) containing 10% fetal bovine serum and 1% penicillin/streptomycin, in a humidified atmosphere of 5% CO_2_ at 37 °C. When cells reached 80% confluence, they were trypsinized and seeded into 96-well plates at a density of 3,000 cells per well. After an overnight incubation, various concentrations of the compounds (NAGN, NH, and SA) were added to the wells. Following a 24-h incubation period, the Cell Counting Kit-8 reagent was introduced into each well. The plates were then incubated at 37 °C for 2 h, after which the absorbance at 450 nm was measured using a microplate reader.

### Fluorescence imaging

#### Cell imaging

A549 and SH-SY5Y cells were seeded into 6-well plates and cultured for 24 h at 37 °C under 5% CO_2_. To determine the optimal probe incubation time, cells were treated with the substrate for varying durations. Subsequently, A549 was cotreated with different concentrations of d-glucose or the OGA-positive inhibitor T-G for 24 h, followed by incubation with 10 μM NAGN for 8 h. After treatment, the cells were washed 3 times with PBS and imaged using a Cytation 5 imaging multimode microplate reader with a 10× objective.

#### Fluorescence imaging in animal tissues

For ex vivo incubation, fresh tissues (kidney, heart, liver, spleen, lung, and brain) were harvested from normal mice and placed in a culture dish. The tissues were incubated with the fluorescent probe NAGN (20 μM) in a PBS-based reaction system for 2 h at 37 °C. Following incubation, the tissues were rinsed 3 times with PBS, immersed in a small volume of PBS for moisture retention, and immediately subjected to fluorescence imaging using a small-animal imaging system (VISQUE InVivo Smart-LF). To investigate the in vivo distribution profile of the probe, mice received an intraperitoneal injection of NAGN (20 μM). At specified time intervals (0.5 and 4 h) postadministration, the mice were sacrificed, and relevant tissues were collected for ex vivo fluorescence imaging.

### siRNA transfection

Cells were seeded into 6-well plates at a density of 5 × 10^5^ cells/well and cultured overnight to reach 60% to 70% confluence. SH-SY5Y cells were transfected with *OGA*-targeting siRNA, while HT-22 and BV2 cells were transfected with *Efnb2*-targeting siRNA using Lipo8000 reagent (Beyotime, China), according to the manufacturer’s instructions. Briefly, 100 pmol of siRNA and 4 μl of Lipo8000 were separately diluted in 125 μl of Opti-MEM medium, gently mixed, and incubated for 20 min at room temperature. The resulting transfection mixture was then added dropwise to the cells. A scrambled siRNA was used as the negative control, and the sequences of all siRNAs are provided in Table S6. Cells were incubated with the transfection mixture for 36 h prior to subsequent experimental treatments and analyses. The efficiency of siRNA transfection was validated by WB.

### Overexpression of WT or mutant EphrinB2

To perform the rescue experiments, *Efnb2*-knockdown BV2 and HT-22 cells were transfected with 5 μg of WT (pCMV-WT-*Efnb2*-HA) or mutant plasmids. Following a 24-h incubation period, the cells were treated with MPP^+^ and with or without SA. Subsequently, the cells were harvested for IP or WB analysis.

### CHX chase assay

To determine the effect of SA on the half-life of EphrinB2, BV2 and HT-22 cells overexpressing WT or mutant EphrinB2 were seeded into 6-well plates. The cells were then treated with SA and CHX for 0, 2, 4, and 8 h, respectively. Subsequently, the cells were harvested and subjected to WB analysis.

### Animal experiments

Male C57BL/6J mice (8 to 10 weeks old) were randomly divided into 4 groups (*n* = 5): control, MPTP (20 mg/kg), MPTP+SA (20 mg/kg), and MPTP+SA (40 mg/kg). After 6-d acclimation, SA or physiological saline was administered daily via intraperitoneal injection from day 7, as outlined in Fig. 5C. Upon treatment completion, mice underwent behavioral assessment in a quiet setting and were subsequently euthanized for tissue analysis. All procedures were approved by the Experimental Animal Care and Use Committee of the Shanghai University of Traditional Chinese Medicine (Approval No. PZSHUTCM2311130003) and conducted in accordance with institutional guidelines.

### Western blot

Cellular and tissue lysates were extracted using radioimmunoprecipitation assay (RIPA) buffer supplemented with phosphatase and protease inhibitors, with the total protein concentration determined by bicinchoninic acid (BCA) assay. Proteins were separated by Tris–glycine sodium dodecyl sulfate–polyacrylamide gel electrophoresis (SDS-PAGE; 10% or 12.5% gels) and transferred to polyvinylidene fluoride membranes. After blocking (protein-free rapid blocking buffer, 30 min), membranes were incubated with primary antibodies overnight at 4 °C, followed by secondary antibodies for 1 h at room temperature. Protein bands were visualized using Bio-Rad ChemiDoc Touch Imaging System with a chemiluminescent substrate and quantified with the ImageJ software. The following primary antibodies were used: anti-active GAPDH (Proteintech, 1:5,000); anti-TH (Proteintech, 1:3,000); anti-*O*-GlcNAc (PTMBIO, 1:500); and anti-p-ERK, anti-ERK, EphrinB2, EphB4 (Immunoway, 1:3,000).

### RNA isolation and qRT-PCR

Total RNA was isolated from 50 to 100 mg of murine brain tissue using the RNApure Tissue & Cell Kit per the manufacturer’s protocol, followed by complementary DNA synthesis with PrimeScript RT Master Mix (Takara). Quantitative polymerase chain reaction analysis was conducted on a QuantStudio 5 thermocycler (Thermo Fisher Scientific) using TB Green Premix Ex Taq, under the following cycling conditions: 95 °C for 30 s, 40 cycles of 95 °C for 5 s, and 60 °C for 30 s. All quantitative real-time polymerase chain reaction (qRT-PCR) primer sequences are listed in Table S7.

### ROS and MMP measurement

The levels of ROS and MMP were detected using 2,7-dichlorodihydrofluorescein diacetate (DCFH-DA) and Rhodamine 123 (Rh123). Cells or mouse brain lysates were incubated with 10 μM DCFH-DA or Rh123 at 37 °C for 30 min. DCFH-DA or Rh123 was removed, and cells were washed 3 times with PBS. Fluorescence was measured using a fluorescence microscope or microplate reader. The intensity of the fluorescence was qualified using ImageJ.

### Tissue biodistribution assessment of SA

To evaluate the bioavailability of SA, 35 mice were administered SA (20 mg/kg) via intraperitoneal injection. Blood samples were collected at 0 to 12 h post-dose (0, 0.5, 1, 2, 4, 8, and 12 h). The serum was separated by centrifugation and deproteinized with 3 volumes of acetonitrile; the supernatant was then subjected to SA quantification. For the tissue distribution study, mice were euthanized at 0.5, 1, 2, 4, and 12 h after SA administration, and tissues (brain, heart, liver, spleen, lung, and kidney) were rapidly excised. After surface moisture was gently blotted, each tissue sample was weighed, homogenized in PBS using grinding beads, and centrifuged. The resulting supernatant was deproteinized with 3 volumes of acetonitrile. SA concentrations in both serum and tissue supernatants were quantified by LC–MS/MS.

For the pharmacokinetic study in PD model mice, the PD model was first established by intraperitoneal injection of MPTP (20 mg/kg daily) for 7 consecutive days. Subsequently, SA (20 mg/kg) was administered intraperitoneally, and blood and tissue samples were collected and processed as described above.

### LC–MS/MS analysis of SA

Samples were analyzed using an LC–MS/MS system composed of a Shimadzu LC-20A liquid chromatograph (Kyoto, Japan) and an Applied Biosystems Sciex Qtrap 4500 mass spectrometer (Massachusetts, USA). Chromatographic separation was performed on an Agilent Poroshell 120 SB-C18 column (2.1 mm × 100 mm, 2.7 μm) with a mobile phase of solvent A (0.1% formic acid in water) and solvent B (acetonitrile). The gradient program was as follows: 0.0 to 1.0 min, 35% to 98% B; 1.0 to 2.5 min, 98% B; 2.5 to 2.6 min, 98% to 35% B; and 2.6 to 4.5 min, 35% B. The flow rate was 0.35 ml/min. Mass spectrometric detection was performed in negative ion switching mode using multiple reaction monitoring (MRM). The ion source temperature was 500 °C, and the ion spray voltage was set to −4,500 V (negative). The nebulizer gas (GAS1) and auxiliary heating gas (GAS2) were both maintained at 30 psi, with the curtain gas at 20 psi; nitrogen served as the gas source. The MRM transition of SA was set at parent ion *m*/*z* 861.2 to fragment ion *m*/*z* 386.0 [52], so the MRM transition for SA was defined as *m*/*z* 861.2 → 386.0 for targeted quantification, declustering potential (DP) −50.0 V, collision energy (CE) −55.0 eV; indomethacin (internal standard), *m*/*z* 356.2 → 312.2, DP −8.0 V, CE −12.0 eV. The Analyst software (AB Sciex, version 1.6.3) was used for data acquisition and peak integration.

### Behavioral tests

#### Open-field test

The open-field test was performed in a white plexiglass arena (40 × 40 × 60 cm). Each mouse was placed in the center of the arena, facing away from the experimenter, after removal from the home cage. Its activity was recorded for a 10-min session using an overhead video camera. The total distance traveled and movement trajectories were subsequently analyzed using the ANY-maze video-tracking-based animal behavior analysis system (Stoelting, USA).

#### Pole test

Mice were acclimatized to the apparatus by being placed at the top of a vertical rough-textured pole (diameter: 1 cm; height: 50 cm) and allowed to descend to the home cage twice. For the formal test, each mouse was placed head-up at the top of the pole, and the times to turn completely downward (T-turning) and to descend to the base (T-climbing) were recorded. Each mouse underwent 2 to 3 trials with a 5-min inter-trial interval, and the average performance across trials was calculated for statistical analysis.

### ELISA for the detection of inflammatory factors

Mouse brain tissues were harvested and rinsed with ice-cold PBS and then homogenized in PBS containing protease inhibitor cocktail. The supernatant was collected as brain S9 fraction and stored at −80 °C. The total protein concentration was determined by BCA assay for data normalization. The levels of TNF-α, IL-6, and IL-10 were detected using commercial mouse ELISA kits following the manufacturer’s protocols. Standard series, sample, and blank control wells were arranged. After incubation at 37 °C and plate washing, enzyme-conjugated secondary antibody was added, followed by chromogenic reaction in the dark. The absorbance at 450 nm was measured with a microplate reader. Cytokine concentrations were calculated according to the standard curve and expressed as picograms per microgram protein.

### Intervention experiment with the EphB4-specific inhibitor

BV2 microglial cells were used to establish an MPP^+^-induced PD cell model, with the EphB4-specific inhibitor NVP-BHG712 used for intervention. Four groups were set: control (DMSO), MPP^+^ model (only MPP^+^ induction), SA intervention (MPP^+^-induced PD model treated with SA), and SA+NVP-BHG712 combined intervention (MPP^+^-induced PD model cotreated with SA and NVP-BHG712). All cells were cultured in DMEM with 10% fetal bovine serum at 37 °C and 5% CO_2_, seeded into 6-well plates at the logarithmic growth phase for 24 h, then subjected to grouped drug intervention, further cultured for 24 h, and harvested for mRNA detection.

### IP assays

Cells were seeded into 10-cm dishes and treated with SA or the vehicle as indicated. After treatment, cells were washed twice with ice-cold PBS and lysed in RIPA buffer (50 mM Tris–HCl, pH 7.4, 150 mM NaCl, 1% NP-40, 0.5% sodium deoxycholate, 0.1% SDS) supplemented with a protease inhibitor cocktail. Cell lysates were sonicated briefly and centrifuged at 12,000 × g for 15 min at 4 °C. The supernatant was collected, and the protein concentration was determined using a BCA assay. For IP, 500 μg of total protein (in a 100-μl lysate volume) was incubated with 2 μg of anti-EphrinB2 antibody overnight at 4 °C with gentle rotation. Protein A/G agarose beads were then added to capture the antigen–antibody complexes for 3 h at 4 °C. The beads were collected and washed 5 times with ice-cold lysis buffer. Finally, the bound proteins were eluted by boiling in 1× SDS-PAGE loading buffer for 10 min and subjected to subsequent WB analysis.

### Hematoxylin and eosin staining and immunohistochemistry staining

Mouse tissues were fixed in 4% formalin, paraffin-embedded, and processed for hematoxylin and eosin staining and immunohistochemistry as previously described. For hematoxylin and eosin staining, paraffin sections (4 to 5 μm) were baked at 65 °C for 1 h, dewaxed in xylene, and rehydrated through a descending ethanol series (100%, 95%, and 70% ethanol). Sections were subjected to hematoxylin staining for a period of 5 to 10 min. Subsequently, they were differentiated in a 1% hydrochloric acid–alcohol solution and rinsed with tap water until a blue hue was achieved. They were then counterstained with eosin for 2 to 5 min. Following dehydration and clearing, sections were coverslipped with neutral balsam and observed by light microscope. For immunohistochemistry staining, dewaxed and rehydrated sections underwent antigen retrieval by heating in 10 mM citrate buffer (pH 6.0) followed by preincubation for 1 h with normal goat serum to block nonspecific binding. Sections were incubated overnight at 4 °C with primary antibodies against TH (Proteintech Group, Inc.) or *O*-GlcNAc (PTMBIO, China). After washing, bound antibodies were detected using appropriate secondary antibodies, followed by visualization with NovaRed or diaminobenzidine chromogen. After counterstaining with hematoxylin, sections were dehydrated, cleared, and mounted.

### Proteomics analysis

#### Protein extraction and digestion

Mouse brain tissues were collected and dissected to isolate the STR on ice and then stored at −80 °C until use. Tissues were homogenized in a cold lysis buffer (8 M urea, 1% protease inhibitor cocktail, and 1% phosphatase inhibitor). The homogenate was centrifuged at 12,000 × g for 15 min at 4 °C, and the supernatant was collected for protein concentration determination by BCA assay. For each sample, an aliquot containing 100 μg of protein was reduced with 10 mM dithiothreitol at 37 °C for 1 h, alkylated with 20 mM iodoacetamide in the dark for 30 min, and then diluted with 50 mM ammonium bicarbonate to reduce the urea concentration to below 1 M. Trypsin was added at a 1:50 (enzyme-to-protein) ratio for overnight digestion at 37 °C. The resulting peptides were desalted using C18 solid-phase extraction cartridges, vacuum-dried, and reconstituted in 0.1% formic acid for LC–MS/MS analysis.

#### DIA-based nano-LC–MS/MS analysis

The digested peptides were separated on a nanoElute UHPLC system (Bruker Daltonics) using a reversed-phase C18 column (25 cm × 75 μm, 1.6-μm particle size). The gradient was set from 2% to 22% acetonitrile (in 0.1% formic acid) over 90 min at a flow rate of 300 nl/min. MS analysis was performed on a timsTOF Pro 2 instrument (Bruker Daltonics) coupled with the nanoElute system. For data-independent acquisition (DIA), a 100-ms trapped ion mobility spectrometry accumulation time and a mass range of 100 to 1,700 *m*/*z* were used. The DIA scheme was designed with variable isolation windows covering the entire *m*/*z* range, and each window was acquired with a CE ramp. A spectral library was constructed from data-dependent acquisition runs of pooled samples, which were processed using MaxQuant (version 2.4.0) against the UniProt mouse database.

#### Data processing and bioinformatics

The DIA raw data were analyzed with Spectronaut (version 18.0) using the previously generated spectral library. Default settings were applied for cross-run normalization, interference correction, and protein identification. Proteins were quantified based on the summed intensity of their top 3 precursor ions, and only proteins with at least 2 unique peptides were retained for downstream analysis. Statistical analysis was performed using the Perseus software (version 2.0.10): proteins with a fold change >1.2 or <0.83 and a *P* value <0.05 (Student *t* test) were considered differentially expressed. Functional annotation and pathway enrichment analysis were conducted with the clusterProfiler package in R, based on Gene Ontology and KEGG databases.

#### Identifying the *O*-GlcNAcylation sites of EphrinB2

Briefly, 50 μg of mouse or human EphrinB2 was incubated with 6 μg of OGT and 500 μM UDP-GlcNAc in a 100-μl reaction buffer (50 mM Tris–HCl, pH 7.4, 12.5 mM MgCl_2_) for 4 h at 37 °C. Following tryptic digestion, the peptides were desalted using C18 solid-phase extraction cartridges and subjected to LC–MS/MS for *O*-GlcNAcylation site mapping. Peptide mixtures were analyzed by an EASY-nLC 1200 nano-LC system coupled to a Q Exactive HF-X Orbitrap mass spectrometer. Peptides were loaded onto a reversed-phase analytical column and separated using a binary solvent system (solvent A: 0.1% formic acid in water; solvent B: 80% acetonitrile with 0.1%) via a linear gradient, followed by high organic phase elution for column washing at a set flow rate. The mass spectrometer was operated in data-dependent acquisition mode. Full MS scans were acquired in the Orbitrap (*m/*z 350 to 1,500, resolution 60,000) with an automatic gain control (AGC) target of 3 × 10^6^ and the specified maximum injection time. The top 15 intense precursor ions were subjected to higher-energy collisional dissociation fragmentation; MS/MS spectra were acquired at a resolution of 15,000 or 30,000, with the specified AGC target, the maximum injection time, and normalized CE, with dynamic exclusion enabled. Raw MS files were processed by the Proteome Discoverer software. MS/MS spectra were searched against a custom FASTA database (mouse/human EphrinB2 protein sequences + common contaminant sequences) using Sequest HT. Trypsin was used with up to 3 missed cleavages; fixed modification: cysteine carbamidomethylation; variable modifications: methionine oxidation, protein N-terminal acetylation, and serine/threonine GlcNAc modification (+203.0794 Da). Precursor/fragment ion mass tolerances were 10 ppm and 0.02 Da, respectively; peptide-spectrum matches were filtered by Percolator (false discovery rate 1%). Candidate EphrinB2 GlcNAc-modified peptides were manually verified based on MS/MS spectral quality, fragment coverage, and diagnostic GlcNAc fragment ions (*m*/*z* 204.0867). *O*-GlcNAcylation site localization was assessed via site-determining fragment ions and ptmRS probability (when available); only high-confidence peptide-spectrum matches with clear site-localizing fragments were considered specific GlcNAc sites.

## Data Availability

All data are available in the main text or the Supplementary Materials.

## References

[1] Li Y, Lv Z, Dai Y, Yu L, Zhang L, Wang K, Hu P. The global, regional, and National burden of parkinson’s disease in 204 countries and territories, 1990–2021: A systematic analysis for the global burden of disease study 2021. BMC Public Health. 2025;25(1):3047. 40940662 10.1186/s12889-025-23492-8 PMC12427116

[2] Tolosa E, Garrido A, Scholz SW, Poewe W. Challenges in the diagnosis of Parkinson’s disease. Lancet Neurol. 2021;20(5):385–397. 33894193 10.1016/S1474-4422(21)00030-2 PMC8185633

[3] Elsworth JD. Parkinson’s disease treatment: Past, present, and future. J Neural Transm (Vienna). 2020;127(5):785–791. 32172471 10.1007/s00702-020-02167-1 PMC8330829

[4] Morris HR, Spillantini MG, Sue CM, Williams-Gray CH. The pathogenesis of Parkinson’s disease. Lancet. 2024;403(10423):293–304. 38245249 10.1016/S0140-6736(23)01478-2

[5] Mirzac D, Glaser MB, Kreis SL, Ringel F, Bange M, Herz DM, Groppa S, Rotaru L, Almeida V, Blech J, et al. Cortical single-cell primers of abnormal brain activity in Parkinson’s disease. Research. 2025;8: Article 0863. 40948939 10.34133/research.0863 PMC12423508

[6] Meng T, Zhang Y, Fu S, Ma S. Gpr35 expression mitigates neuroinflammation and enriches gut *Lactobacillus* to relieve Parkinson’s disease. Research. 2025;8:0846. 40862055 10.34133/research.0846 PMC12376290

[7] Kim TY, Lee BD. Current therapeutic strategies in Parkinson’s disease: Future perspectives. Mol Cells. 2025;48: Article 100274. 40914459 10.1016/j.mocell.2025.100274 PMC12481064

[8] Stocchi F, Bravi D, Emmi A, Antonini A. Parkinson disease therapy: Current strategies and future research priorities. Nat Rev Neurol. 2024;20(12):695–707. 39496848 10.1038/s41582-024-01034-x

[9] Kulkarni SR, Thokchom B, Abbigeri MB, Bhavi SM, Singh SR, Metri N, Yarajarla RB. The role of L-DOPA in neurological and neurodegenerative complications: A review. Mol Cell Biochem. 2025;480(10):5221–5242. 40488810 10.1007/s11010-025-05324-w

[10] Yang X, Qian K. Protein *O*-GlcNAcylation: Emerging mechanisms and functions. Nat Rev Mol Cell Biol. 2017;18(7):452–465. 28488703 10.1038/nrm.2017.22 PMC5667541

[11] Hou C, Li W, Li Y, Ma J. *O*-GlcNAc informatics: Advances and trends. Anal Bioanal Chem. 2025;417(5):895–905. 39294469 10.1007/s00216-024-05531-2

[12] Qi B, Chen Y, Chai S, Lu X, Kang L. O-linked β-N-acetylglucosamine (O-GlcNAc) modification: Emerging pathogenesis and a therapeutic target of diabetic nephropathy. Diabet Med. 2025;42(2): Article e15436. 39279604 10.1111/dme.15436 PMC11733667

[13] Balana AT, Mahul-Mellier A-L, Nguyen BA, Horvath M, Javed A, Hard ER, Jasiqi Y, Singh P, Afrin S, Pedretti R, et al. O-GlcNAc forces an α-synuclein amyloid strain with notably diminished seeding and pathology. Nat Chem Biol. 2024;20(5):646–655. 38347213 10.1038/s41589-024-01551-2 PMC11062923

[14] Nagel AK, Ball LE. O-GlcNAc transferase and O-GlcNAcase: Achieving target substrate specificity. Amino Acids. 2014;46(10):2305–2316. 25173736 10.1007/s00726-014-1827-7 PMC4584397

[15] Li Q, Cheng Y, Yang C, Tian M, Wang X, Li D, Li X, Qu J, Zhou S, Zheng L, et al. Targeting the exonic circular *OGT* RNA/O-GlcNAc transferase/forkhead box C1 axis inhibits asparagine- and alanine-mediated ferroptosis repression in neuroblastoma progression. Research. 2025;8: Article 0703. 40416363 10.34133/research.0703 PMC12099056

[16] Yuzwa SA, Shan X, Jones BA, Zhao G, Woodward ML, Li X, Zhu Y, McEachern EJ, Silverman MA, Watson NV, et al. Pharmacological inhibition of *O*-GlcNAcase (OGA) prevents cognitive decline and amyloid plaque formation in bigenic tau/APP mutant mice. Mol Neurodegener. 2014;9(1):42. 25344697 10.1186/1750-1326-9-42 PMC4232697

[17] Keembiyehetty C, Love DC, Harwood KR, Gavrilova O, Comly ME, Hanover JA. Conditional knock-out reveals a requirement for *O*-linked *N*-acetylglucosaminase (*O*-GlcNAcase) in metabolic homeostasis. J Biol Chem. 2015;290(11):7097–7113. 25596529 10.1074/jbc.M114.617779 PMC4358131

[18] Whitworth GE, Macauley MS, Stubbs KA, Dennis RJ, Taylor EJ, Davies GJ, Greig IR, Vocadlo DJ. Analysis of PUGNAc and NAG-thiazoline as transition state analogues for human *O*-GlcNAcase: Mechanistic and structural insights into inhibitor selectivity and transition state poise. J Am Chem Soc. 2007;129(3):635–644. 17227027 10.1021/ja065697o

[19] Meade J, Mesa H, Alamgir S, Bieniecka I, Liu L, Zhang Q. Synaptic toxicity of OGA inhibitors and the failure of ceperognastat. J Prev Alzheimers Dis. 2026;13: Article 100456. 41478829 10.1016/j.tjpad.2025.100456 PMC12869045

[20] Groves JA, Zachara NE. Characterization of tools to detect and enrich human and mouse *O*-GlcNAcase. Glycobiology. 2017;27:791–795. 28595377 10.1093/glycob/cwx051 PMC5881776

[21] Jin Q, Wu J, Wu Y, Li H, Finel M, Wang D, Ge G. Optical substrates for drug-metabolizing enzymes: Recent advances and future perspectives. Acta Pharm Sin B. 2022;12(3):1068–1099. 35530147 10.1016/j.apsb.2022.01.009 PMC9069481

[22] Wei G, Zhang Z, Wang B, Jin Q, Li X, Jin Z, Liu Y, Zou L, Ge G. Spatiotemporal visualization of prolyl endopeptidase in living systems by an enzyme-activatable NIR fluorogenic probe. Sensors Actuators B Chem. 2026;458: Article 139851.

[23] Alteen MG, Tan HY, Vocadlo DJ. Monitoring and modulating *O*-GlcNAcylation: Assays and inhibitors of *O*-GlcNAc processing enzymes. Curr Opin Struct Biol. 2021;68:157–165. 33535148 10.1016/j.sbi.2020.12.008

[24] Morsby JJ, Smith BD. Advances in optical sensors of *N*-acetyl-β-d-hexosaminidase (*N*-acetyl-β-d-glucosaminidase). Bioconjug Chem. 2022;33(4):544–554. 35302753 10.1021/acs.bioconjchem.2c00057 PMC9870670

[25] Boo J, Lee J, Kim Y-H, Lee C-H, Ku B, Shin I. A fluorescent probe to simultaneously detect both O-GlcNAcase and phosphatase. Front Chem. 2023;11:1133018. 36936532 10.3389/fchem.2023.1133018 PMC10015443

[26] Wu Y-H, Wang G-J, Guo C, Wang P-P, Wang J-Y, Hu X-L, Zang Y, James TD, Li J, He X-P. Isoindoline-based fluorogenic probes bearing a self-immolative linker for the sensitive and selective detection of O-GlcNAcase activity. Chem Commun (Camb). 2024;60(63):8240–8243. 39007923 10.1039/d4cc02845g

[27] Atanasov AG, Zotchev SB, Dirsch VM, International Natural Product Sciences Taskforce, Supuran CT. Natural products in drug discovery: Advances and opportunities. Nat Rev Drug Discov. 2021;20(3):200–216. 33510482 10.1038/s41573-020-00114-z PMC7841765

[28] Shang X, Dai L, Cao X, Ma Y, Gulnaz I, Miao X, Li X, Yang X. Natural products in antiparasitic drug discovery: Advances, opportunities and challenges. Nat Prod Rep. 2025;42(9):1419–1458. 40501439 10.1039/d5np00007f

[29] Mohammadipour A. A focus on natural products for preventing and cure of mitochondrial dysfunction in Parkinson’s disease. Metab Brain Dis. 2022;37(4):889–900. 35156154 10.1007/s11011-022-00931-8

[30] Calvelo M, Males A, Alteen MG, Willems LI, Vocadlo DJ, Davies GJ, Rovira C. Human *O*-GlcNAcase uses a preactivated boat-skew substrate conformation for catalysis. Evidence from X-ray crystallography and QM/MM metadynamics. ACS Catal. 2023;13(20):13672–13678. 37969138 10.1021/acscatal.3c02378 PMC10636738

[31] Hu C-W, Wang A, Fan D, Worth M, Chen Z, Huang J, Xie J, Macdonald J, Li L, Jiang J. OGA mutant aberrantly hydrolyzes O-GlcNAc modification from PDLIM7 to modulate p53 and cytoskeleton in promoting cancer cell malignancy. Proc Natl Acad Sci USA. 2024;121(24): Article e2320867121. 38838015 10.1073/pnas.2320867121 PMC11181094

[32] Videira PAQ, Castro-Caldas M. Linking glycation and glycosylation with inflammation and mitochondrial dysfunction in Parkinson’s disease. Front Neurosci. 2018;12:381. 29930494 10.3389/fnins.2018.00381 PMC5999786

[33] Brockhausen I, Schutzbach J, Wang J, Fishwick B, Brockhausen J. Glycoconjugate journal special issue on: The glycobiology of Parkinson’s disease. Glycoconj J. 2022;39:55–74. 34757539 10.1007/s10719-021-10024-w

[34] Johnson ME, Salvatore MF, Maiolo SA, Bobrovskaya L. Tyrosine hydroxylase as a sentinel for central and peripheral tissue responses in Parkinson’s progression: Evidence from clinical studies and neurotoxin models. Prog Neurobiol. 2018;165:1–25. 29331395 10.1016/j.pneurobio.2018.01.002

[35] Yeung YT, Aziz F, Guerrero-Castilla A, Arguelles S. Signaling pathways in inflammation and anti-inflammatory therapies. Curr Pharm Des. 2018;24(14):1449–1484. 29589535 10.2174/1381612824666180327165604

[36] Qin X, Wang S, Huang J, Hu B, Yang X, Liang L, Zhou R, Huang W. Rhein alleviates MPTP-induced Parkinson’s disease by suppressing neuroinflammation via MAPK/IκB pathway. Front Neurosci. 2024;18:1396345. 38933815 10.3389/fnins.2024.1396345 PMC11202316

[37] Soundararajan P, Fawcett JP, Rafuse VF. Guidance of postural motoneurons requires MAPK/ERK signaling downstream of fibroblast growth factor receptor 1. J Neurosci. 2010;30(19):6595–6606. 20463222 10.1523/JNEUROSCI.4932-09.2010 PMC6632572

[38] Miao H, Wei BR, Peehl DM, Li Q, Alexandrou T, Schelling JR, Rhim JS, Sedor JR, Burnett E, Wang B. Activation of EphA receptor tyrosine kinase inhibits the Ras/MAPK pathway. Nat Cell Biol. 2001;3(5):527–530. 11331884 10.1038/35074604

[39] Jha SK, Jha NK, Kar R, Ambasta RK, Kumar P. p38 MAPK and PI3K/AKT signalling cascades in Parkinson’s disease. Int J Mol Cell Med. 2015;4(2):67–86. 26261796 PMC4499569

[40] Bohush A, Niewiadomska G, Filipek A. Role of mitogen activated protein kinase signaling in Parkinson’s disease. Int J Mol Sci. 2018;19(10):2973. 30274251 10.3390/ijms19102973 PMC6213537

[41] Lu S, Ji N, Wang W, Lin X, Gao D, Geng D. Salidroside improves cognitive function in Parkinson’s disease via Braf-mediated mitogen-activated protein kinase signaling pathway. Biomed Pharmacother. 2024;177: Article 116968. 38901199 10.1016/j.biopha.2024.116968

[42] Martiny-Baron G, Holzer P, Billy E, Schnell C, Brueggen J, Ferretti M, Schmiedeberg N, Wood JM, Furet P, Imbach P. The small molecule specific EphB4 kinase inhibitor NVP-BHG712 inhibits VEGF driven angiogenesis. Angiogenesis. 2010;13(3):259–267. 20803239 10.1007/s10456-010-9183-z PMC2941628

[43] Chen Y, Luo X, Yin Y, Thomas ER, Liu K, Wang W, Li X. The interplay of iron, oxidative stress, and α-synuclein in Parkinson’s disease progression. Mol Med. 2025;31(1):154. 40287631 10.1186/s10020-025-01208-3 PMC12034127

[44] Ng GYQ, Loh ZW-L, Fann DY, Mallilankaraman K, Arumugam TV, Hande MP. Role of mitogen-activated protein (MAP) kinase pathways in metabolic diseases. Genome Integr. 2024;15: Article e20230003. 38770527 10.14293/genint.14.1.004 PMC11102075

[45] Gui C, Ren Y, Chen J, Wu X, Mao K, Li H, Yu H, Zou F, Li W. p38 MAPK-DRP1 signaling is involved in mitochondrial dysfunction and cell death in mutant A53T α-synuclein model of Parkinson’s disease. Toxicol Appl Pharmacol. 2020;388: Article 114874. 31881179 10.1016/j.taap.2019.114874

[46] Chen J, Ren Y, Gui C, Zhao M, Wu X, Mao K, Li W, Zou F. Phosphorylation of Parkin at serine 131 by p38 MAPK promotes mitochondrial dysfunction and neuronal death in mutant A53T α-synuclein model of Parkinson’s disease. Cell Death Dis. 2018;9(6):700. 29899409 10.1038/s41419-018-0722-7 PMC5999948

[47] Wang L, Tian S, Ruan S, Wei J, Wei S, Chen W, Hu H, Qin W, Li Y, Yuan H, et al. Neuroprotective effects of cordycepin on MPTP-induced Parkinson’s disease mice via suppressing PI3K/AKT/mTOR and MAPK-mediated neuroinflammation. Free Radic Biol Med. 2024;216:60–77. 38479634 10.1016/j.freeradbiomed.2024.02.023

[48] Yang J, Jia M, Zhang X, Wang P. Calycosin attenuates MPTP-induced Parkinson’s disease by suppressing the activation of TLR/NF-κB and MAPK pathways. Phytother Res. 2019;33(2):309–318. 30421460 10.1002/ptr.6221

[49] Wu Y-K, Liu M, Zhou H-L, He X, Wei J, Hua W-H, Li H-J, Yuan Q-H, Xie Y-F. O-linked β-N-acetylglucosamine transferase regulates macrophage polarization in diabetic periodontitis: *In vivo* and *in vitro* study. World J Diabetes. 2025;16(2):95092. 40093279 10.4239/wjd.v16.i3.95092 PMC11885980

[50] Lyu C, Yuan L, Yang Y, Zhang D, Hu W, Zhao K, Ding Y, Chen W, Xiao K, Chen Y, et al. Ligand preference of EphB2 receptor is selectively regulated by N-glycosylation. iScience. 2025;28(5): Article 112386. 40330885 10.1016/j.isci.2025.112386 PMC12052844

[51] Gadsbøll N, Høilund-Carlsen PF, Badsberg JH, Lønborg-Jensen H, Stage P, Marving J, Jensen BH. Left ventricular peak filling rate, heart failure and 1-year survival in patients with acute myocardial infarction. Eur Heart J. 1991;12(2):194–202. 2044553 10.1093/oxfordjournals.eurheartj.a059868

[52] Ye M, Han J, Chen H, Zheng J, Guo D. Analysis of phenolic compounds in rhubarbs using liquid chromatography coupled with electrospray ionization mass spectrometry. J Am Soc Mass Spectrom. 2007;18(1):82–91. 17029978 10.1016/j.jasms.2006.08.009

