# Supplementary material for: Discovery of Sennoside A as a Natural O-GlcNAcase Inhibitor That Ameliorates Parkinson’s Disease by Restoring EphrinB2 O-GlcNAcylation
Source: Research (Wash D C). 2026 Sep 9;9:1416. doi: 10.34133/research.1416 (PMC13554434; doi:10.34133/research.1416)
Supplement: Supplementary file 1 [file research.1416.f1.docx]

**Supplementary Materials**

*for*

**Discovery of Sennoside A as a Natural OGA Inhibitor ameliorate Parkinson's Disease by Restoring EphrinB2 *O*-GlcNAcylation**

Guilin Wei^1,2^, Zhuoan Jin^1^, Hanying Mi^1^, Nan Li^1^, Weiyu Wang^1^, Yunqing Song^1^, Qiang Jin^1,^*, Guangbo Ge^1,3,^*, and Yong Liu^2,^*

^1^ State Key Laboratory of Discovery and Utilization of Functional Components in Traditional Chinese Medicine; Shanghai Frontiers Science Center of TCM Chemical Biology; Institute of Interdisciplinary Integrative Medicine Research, Shanghai University of Traditional Chinese Medicine, Shanghai, 201203 China.

^2^ School of Chemical Engineering, Ocean and Life Sciences, Dalian University of Technology, Panjin 124221, China.

^3^Jiangxi Provincial Key Laboratory of Natural Microbial Medicine Research; School of Pharmacy, Yichun University, Yichun, 336000, China.

*Corresponding authors:

E-mail: jindicp@163.com (Qiang Jin); geguangbo@shutcm.edu.cn (Guangbo Ge); yliu@dlut.edu.cn (Yong Liu)

**This file contains 34 supplementary figures and 7 supplementary tables.****Fig. S1** ^1^H NMR spectrum of **NB** in CDCl_3_.

**Fig. S2** ^13^C NMR spectrum of **NB** in CDCl_3_.

**Fig. S3** HRMS spectrum of **NB**.

**Fig. S4** ^1^H NMR spectrum of **NCO** in CDCl_3_.

**Fig. S5** ^13^C NMR spectrum of **NCO** in CDCl_3_.

**Fig. S6** HRMS spectrum of **NCO**.

**Fig. S7** ^1^H NMR spectrum of **NH** in DMSO-d6.

**Fig. S8** ^13^C NMR spectrum of **NH** in DMSO-d6.

**Fig. S9** HRMS spectrum of **NH**.

**Fig. S10** ^1^H NMR spectrum of **NAGN** in DMSO-d6.

**Fig. S11** ^13^C NMR spectrum of **NAGN** in DMSO-d6.

**Fig. S12** HRMS spectrum of **NAGN**.

**Fig. S13** HPLC-UV spectra of (A) **NCO**, purity: 85.33%; (B) **NH**, purity:97.98% and (C) **NAGN**, purity: 96.56%.

**Fig. S14** **Spectroscopic characterization of NAGN.** (A, B) Absorption and fluorescence spectra of **NAGN** in the presence or absence of OGA; (C, D) Fluorescence spectra and fluorescence intensity of **NAGN** upon addition of increasing concentrations of OGA; (E, F) Fluorescence intensity of **NAGN** at different time points after OGA addition. Data were expressed as mean ± SD (n = 3).

**Fig. S15** **Selectivity of the probe towards OGA**. (A) Impact of amino-acid composition and ionic milieu on the catalytic activity of OGA; (B) pH-dependent fluorescence response profile of NH and NAGN; (C) Kinetic plots of OGA-catalyzed **NAGN** hydrolysis obtained from the microplate reader‑based assay; (D) Kinetic plots of HLC-catalyzed **NAGN** hydrolysis obtained from the microplate reader‑based assay; (E) Kinetic plots of OGA-catalyzed **NAGN** hydrolysis from HPLC-UV‑based method. Data were expressed as mean ± SD (n = 3).

**Fig. S16** **Cellular safety of probe substrate NAGN and its hydrolytic product NH.** The cell viability assays of both (A) **NAGN** and its product (B) **NH** in the CTC, BESA-2B, A549, HT-22, and SH-SY5Y cell lines. Data were expressed as mean ± SD (n = 3).

**Fig. S17** **Fluorescence imaging of OGA in living cells**. (A)The optimization of incubation time for **NAGN** (10 μM) in different cell lines, scale bar = 30 μm; (B) The fluorescence quantification graph of Fig. A; (C) Fluorescence imaging of OGA with the addition of T-G (100 μM) and D-glu (10, 20, 40 mM) in A549 cells, scale bar = 30 μm; (D) Quantification graph of Fig. C. Data were expressed as mean ± SD, n = 3, comparisons were analyzed by one-way analysis of variance with Dunnett’s tests. ns, *P* > 0.05; **P* < 0.05; *****P* < 0.001.

**Fig. S18 Fluorescence imaging of OGA in SH-SY5Y cells and siRNA transfected SH-SY5Y cells.** (A) The protein level expression of OGA in SH-SY5Yand siRNA transfected SH-SY5Ycells. (B) Fluorescence images of living SH-SY5Y cells, scale bar = 30 μm.

**Fig. S19** ***In situ* imaging of OGA activities in various organs**. (A) *In situ* imaging of different organs treated with **NAGN** (20 μM); (B) Fluorescence quantification (left) and HPLC-UV quantification (right) of different tissues; *In situ* (C) imaging and (D) fluorescence quantitative of OGA activity levels in multiple organs from mice at 30 min and 4 h post-intraperitoneal injection of NAGN (20 μM). Data were expressed as mean ± SD, n = 3.

**Fig. S20** Chemical structures of Thiamet G (a positive OGA inhibitor) and four naturally derived compounds with potent OGA inhibitory activity.

**Fig. S21 Inhibitory effects of echinocystic acid and SA on OGA.** (A) Dose-inhibition curve of echinocystic acid on OGA. (B) The inhibitory effects of SA (20 μM) towards OGA and other human hydrolases. (C) Time-dependent inhibition curve of SA on OGA. (D) Inhibition kinetics of SA against OGA. Data were expressed as mean ± SD, n = 3.

**Fig. S22** The RMSD fluctuation of SA in the two catalytic sites (cavity A and cavity B) of the OGA homodimer.

**Fig. S23 Cytotoxicity and anti-neurotoxicity of SA.** (A)The cell viability of SA in SH-SY5Y cell lines. (B)The cell viability of SH-SY5Y cell lines challenged with MPP^+^ in the presence or absence of SA. Data were expressed as mean ± SD, n = 3, comparisons were analyzed by one-way analysis of variance with Dunnett’s tests. ns, *P* > 0.05; ***P* < 0.01; ***P < 0.005.

**Fig. S24 Inflammatory responses in BV2 cells challenged with MPP⁺ with or without SA.** mRNA expression of (A) IL-6, (B) TNF-a, and (C) IL-10 in BV2 cell lines treated with MPP^+^

(250, 500 μM) in the presence or absence of SA (10, 20 μM). Data were expressed as mean ± SD (n = 3), comparisons were analyzed by one-way analysis of variance with Dunnett’s tests. ns, *P* > 0.05; **P* < 0.05; ****P* < 0.005, *****P* < 0.001.

**Fig.S25** **Tissue distribution of SA in normal mice and MPTP-challenged mice *via* intraperitoneal injection of SA (20 mg/kg).** (A) SA content in mouse tissues in normal mice; (B) SA content in MPTP challenged mice. Data were expressed as mean ± SD (n = 5).

**Fig. S26** (A) *In situ* imaging of OGA activity in multiple organs with or without SA (20 μM) treatment; (B) Quantitative plot of Fig. A. Data were expressed as mean ± SD (n = 3).

**Fig. S27** (A) *In situ* imaging of brain tissues from different groups of mice pretreated with or without SA (20 or 40 mg/kg) by using NAGN (20 μM) as a fluorogenic probe; (B) Quantitative plot of Fig. A. Data were expressed as mean ± SD (n = 3), comparisons were analyzed by one-way analysis of variance with Dunnett’s tests. *****P* < 0.001.

**Fig. S28** Morphological alterations of major organs in mice following intraperitoneal injection of SA (20 or 40 mg/kg).

**Fig. S29** Representative hematoxylin and eosin (H&E)-stained images of mice organs from different groups.

**Fig. S30 SA elevated the levels of anti-inflammatory cytokine IL-10 in mouse brain.** (A) mRNA and (B) protein levels of IL-10 of mouse brain challenged with MPTP (20 mg/kg) in the presence or absence of SA (20 or 40 mg/kg). Data were expressed as mean ± SD, n = 5, comparisons were analyzed by one-way analysis of variance with Dunnett’s tests. ns, *P*> 0.05; **P* < 0.05, ****P* < 0.005, *****P* < 0.001.

**Fig. S31** **Heatmaps showed the differentially expression proteins in mouse brain.** (A) Heatmaps showed the differentially expression proteins in MPTP vs Control groups; (B) Heatmaps showed the differentially expression proteins in SA-treated groups vs MPTP groups.

**Fig. S32** (A) Volcano plot showed the differentially expression proteins (DEPs) in MPTP vs Control groups; (B) Volcano plot showed the differentially expression proteins (DEPs) in SA-treated vs MPTP groups.

**Fig. S33 GO analysis of proteomics data following treatment with SA.**

**Fig. S34 The anti-inflammatory effects of SA in BV2 cells treated with EphB4 inhibitor NVP-BHG712.**

**Table S1.** The predicted catalytic distances between each substrate candidate and the N/D‑175 residue within the OGA catalytic cavity across different crystal structures.

| **Substrate** | **Catalytic Distances (Å)** | | | | |
| --- | --- | --- | --- | --- | --- |
|  | 5VVO | 5VVT | 5VVU | 5M7U | 5TKE |
| 1 | 11.2 | 7.5 | 8.1 | 6.3 | 8.9 |
| 2 | 5.8 | 7.1 | 3.8 | 9 | 8.6 |
| 3 | 8.5 | 10.6 | 10 | 9.1 | 3.8 |
| 4 | 7.4 | 5.1 | 6.3 | 10.8 | 8.2 |
| 5 | 8.4 | 9.2 | 11.6 | 3.8 | 11.9 |
| 6 | 8.8 | 8.8 | 7.2 | 4.4 | 4 |
| 7 | 7.2 | 6.1 | 6.3 | 12 | 11 |
| 8 | 7.8 | 8.6 | 7.8 | 7.7 | 7.6 |
| 9 | 8.7 | 11.6 | 10.4 | 5.5 | 6.6 |
| 10 | 7.9 | 5.6 | 7.8 | 11.7 | 11.1 |
| 11 | 11.1 | 5.9 | 11.6 | 7.5 | 6.2 |
| 12 | 13.9 | 9.1 | 5.2 | 9.9 | 6.2 |
| 13 | 4.6 | 6 | 5.7 | 11.3 | 15.1 |
| 14 | 4.8 | 13.5 | 16.6 | 13.7 | 12.8 |
| 15 | 7.4 | 8.6 | 4.9 | 5.4 | 14.4 |
| 16 | 7.9 | 8.3 | 11.2 | 13.7 | 10.8 |
| 17 | 5.9 | 7.2 | 11.2 | 6.9 | 14 |
| 18 | 7.5 | 14.9 | 14.3 | 7.4 | 13.8 |
| 19 | 5.8 | 7.9 | 10.2 | 6.6 | 9 |
| 20 | 6.8 | 7.9 | 6.5 | 6.4 | 6.3 |
| 21 | 4.8 | 5.1 | 4 | 5.9 | 5.3 |
| 22 | 8.8 | 6.5 | 15.9 | 5.8 | 9.4 |
| 23 | 5 | 5.2 | 3.9 | 5.7 | 4.2 |

**Table S2.** The predicted binding affinity (kcal^-1^) of various naphthalimide derivatives on OGA (PDB ID: 5VVO).

| **Naphthalimide derivatives** | **Distance(Å)** |
| --- | --- |
| 1 | -9.1 |
| 2 | -8.6 |
| 3 | -9 |
| 4 | -8.9 |
| 5 | -8.1 |
| 6 | -8.9 |
| 7 | -8.9 |
| 8 | -8.9 |
| 9 | -8.5 |
| 10 | -8.6 |

**Table S3** Enzyme kinetic parameters of NAGN with OGA and HLC.

| enzyme sources | Method | *V*_max_ (nmol·min^−1^·mg protein^−1^) | *K*_m_ (μM) | CL_int_ (μL·min^−1^·mg protein^−1^) |
| --- | --- | --- | --- | --- |
| OGA | Microplate | 22.10 ± 1.18 | 70.47 ± 11.41 | 313.61 |
|  | HPLC-UV | 18.73 ± 1.17 | 62.52 ± 14.08 | 299.58 |
| HLC | Microplate | 0.8157 ± 0.0369 | 85.57 ± 9.65 | 9.533 |

**Table S4** IC₅₀ values of Thiamet G against OGA using different assay methods.

| **IC₅₀ (**nM**)** | |
| --- | --- |
| Microplate based method | HPLC-UV based method |
| 2.98 ± 0.70 | 2.05 ± 0.48 |

**Table S5.** The residual activity of each tested compound against OGA.

| **No.** | **Compound name** | **CAS** | **Residual activity (%)** |
| --- | --- | --- | --- |
|  |  |  | **20 μM** |
| A1 | Control | - | 100.00 |
| A2 | Thiamet-G | 1009816-48-1 | 18.67 |
| A3 | 2',4'-Dihydroxychalcone | 1776-30-3 | 79.29 |
| A4 | 2'-Hyhroxy-4'-methoxychalcone | 39273-61-5 | 69.00 |
| A5 | Chalcone | 94-41-7 | 75.78 |
| A6 | Chrysophal 8-O-glucoside | 13241-28-6 | 137.74 |
| A7 | Emodin-3-methyl ether | 521-61-9 | 57.38 |
| A8 | Emodin | 518-82-1 | 74.71 |
| A9 | Sennoside A | 81-27-6 | 11.32 |
| A10 | Paeonol | 552-41-0 | 102.08 |
| A11 | Paeonol protoside | 72520-92-4 | 98.82 |
| A12 | Salvia lactone | 5957-80-2 | 83.90 |
| B1 | Magnolol | 528-43-8 | 100.38 |
| B2 | Protocatechualdehyde | 139-85-5 | 137.45 |
| B3 | Chlorogenic acid | 327-97-9 | 61.95 |
| B4 | Ethyl gallate | 831-61-8 | 125.55 |
| B5 | Protocatechuic acid | 99-50-3 | 118.13 |
| B6 | 2,5-Dihydroxybenzoic acid | 490-79-9 | 90.34 |
| B7 | Praeruptorin A | 73069-25-7 | 91.92 |
| B8 | baohuoside I | 113558-15-9 | 108.46 |
| B9 | Kaempferol | 520-18-3 | 89.95 |
| B10 | Cianidanol | 154-23-4 | 132.33 |
| B11 | Astragalus glycoside | 480-10-4 | 107.19 |
| B12 | Nobiletin | 478-01-3 | 60.48 |
| C1 | Hesperidin | 520-26-3 | 68.54 |
| C2 | 3,7-Dihydroxyflavone | 492-00-2 | 80.28 |
| C3 | Sciadopitysin | 521-34-6 | 86.83 |
| C4 | Luteolin | 491-70-3 | 107.96 |
| C5 | Quercetin | 117-39-5 | 32.56 |
| C6 | Bilobetin | 521-32-4 | 96.88 |
| C7 | Dihydromyricetin | 27200-12-0 | 97.69 |
| C8 | Chrysin | 480-40-0 | 96.86 |
| C9 | Cynaroside | 5373-11-5 | 69.46 |
| C10 | Glycitein | 40957-83-3 | 104.39 |
| C11 | Quercetin 3-O-β-D-glucofuranoside | 21637-25-2 | 70.33 |
| C12 | Quercitrin | 522-12-3 | 77.33 |
| D1 | Neohesperidin dihydrochalcone | 20702-77-6 | 104.92 |
| D2 | Hesperetin | 520-33-2 | 93.17 |
| D3 | Linarin | 480-36-4 | 87.50 |
| D4 | Neohesperidin | 13241-33-3 | 95.96 |
| D5 | Morin | 480-16-0 | 52.45 |
| D6 | 3,6-Dihydroxyflavone | 108238-41-1 | 91.35 |
| D7 | Flavonol | 577-85-5 | 105.52 |
| D8 | Silybin A | 22888-70-6 | 97.82 |
| D9 | 7,8-Dihydroxyflavone | 38183-03-8 | 87.57 |
| D10 | 4′-Hydroxyflavone | 4143-63-9 | 78.86 |
| D11 | 5,7,8-Trihydroxyflavone | 4443-09-8 | 56.28 |
| D12 | Flavone | 525-82-6 | 50.38 |
| E1 | 3-Methoxyflavone | 7245-02-5 | 67.85 |
| E2 | Isovitexin | 38953-85-4 | 89.33 |
| E3 | Nicotiflorin | 17650-84-9 | 99.26 |
| E4 | Icaritin | 118525-40-9 | 115.36 |
| E5 | Corosolic acid | 4547-24-4 | 105.52 |
| E6 | Palmatine | 3486-67-7 | 126.75 |
| E7 | Asiatic acid | 464-92-6 | 95.27 |
| E8 | Oleanolic Acid | 508-02-1 | 57.48 |
| E9 | Oleanonic acid | 17990-42-0 | 71.91 |
| E10 | Benzoylpaeoniflorin | 38642-49-8 | 119.68 |
| E11 | Polyphyllin I | 50773-41-6 | 144.23 |
| E12 | Formosanin C | 50773-42-7 | 107.39 |
| F1 | Polyphyllin VI | 55916-51-3 | 166.46 |
| F2 | Mudanpioside C | 172760-03-1 | 64.66 |
| F3 | Ursolic acid | 77-52-1 | 85.18 |
| F4 | Echinocystic Acid | 510-30-5 | 36.16 |
| F5 | Sanguinarine | 5578-73-4 | 109.19 |
| F6 | Palmatine chloride | 10605-02-4 | 129.85 |
| F7 | Monocrotaline | 315-22-0 | 120.57 |
| F8 | Tetrahydroberberine | 522-97-4 | 129.08 |
| F9 | Rutaecarpine | 84-26-4 | 102.45 |
| F10 | Leonurine | 24691-74-3 | 107.46 |
| F11 | Berberine chloride | 633-65-8 | 113.28 |
| F12 | Coptisine chloride | 6020-18-4 | 102.94 |
| G1 | Dehydrocorydaline | 30045-16-0 | 94.26 |
| G2 | Nuciferine | 475-83-2 | 125.20 |
| G3 | Jatrorrhizine | 3621-38-3 | 114.91 |
| G4 | Lycorine hydrochloride | 2188-68-3 | 107.66 |
| G5 | Matrine | 519-02-8 | 97.21 |
| G6 | Phellodendrine chloride | 104112-82-5 | 112.05 |
| G7 | Strychnine | 57-24-9 | 116.58 |
| G8 | Tetrahydropalmatine | 2934-97-6 | 113.20 |
| G9 | Reserpine | 50-55-5 | 146.06 |
| G10 | Dendrobine | 2115-91-5 | 98.47 |
| G11 | Evodiamine | 518-17-2 | 112.41 |
| G12 | Dauricine | 524-17-4 | 136.56 |
| H1 | Matrine | 519-02-8 | 126.81 |
| H2 | Testosterone decanoate | 5721-91-5 | 83.98 |
| H3 | Ergosterol | 57-87-4 | 113.36 |
| H4 | Testosterone propionate | 57-85-2 | 127.38 |
| H5 | Genistein | 446-72-0 | 87.92 |
| H6 | Polydatin | 65914-17-2 | 99.38 |
| H7 | Acetylshikonin | 24502-78-1 | 27.63 |

Table S6. Sequence for the siRNAs used in this study.

| **Gene** | **Sense strand (5′→3′)** | **Antisense strand (5′→3′)** |
| --- | --- | --- |
| siRNA-*OGA*1 | GUGAAAUUGUUGAAGCGAATT | UUCGCUUCAACAAUUUCACTT |
| siRNA-*OGA*2 | GCACUUACUUGCUGAUCUATT | UAGAUCAGCAAGUAACUGCTT |
| siRNA-NC | UUCUCCGAACGUGUCACGUTT | ACGUGACACGUUCGGAGAATT |
| siRNA-*Efnb2* | GGUAUUAGGUACAGAAGAATT | UUCUUCUGUACCUAAUACCTT |

**Table S7.** Primer sequence for qRT-PCR.

| **Gene** | **Forward primer (5′ → 3′)** | **Reverse primer (5′ → 3′)** |
| --- | --- | --- |
| m-*Gapdh* | TGATGGGTGTGAACCACGAG | TGCAGGGATGATGTTCTGGG |
| m-*IL-6* | TTCCATCCAGTTGCCTTCTT | CAGAATTGCCATTGCACAAC |
| m-*TNF-a* | ATGAGCACAGAAAGCATGATC | TACAGGCTTGTCACTCGAATT |
| m-*IL-10* | TTCTTTCAAACAAAGGACCAGC | GCAACCCAAGTAACCCTTAAAG |
